# *Ppp4r3a* deficiency leads to depression-like behaviors in mice by modulating the synthesis of synaptic proteins

**DOI:** 10.1242/dmm.049374

**Published:** 2022-05-20

**Authors:** Fei Gao, Ai Liu, Xing Qi, Meitian Wang, Xiao Chen, Shijun Wei, Shang Gao, Yueqing Sun, Ping Sun, Xi Li, Wenjie Sun, Jiangxia Li, Qiji Liu

**Affiliations:** Key Laboratory for Experimental Teratology of the Ministry of Education and Department of Medical Genetics, School of Basic Medical Sciences, Cheeloo College of Medicine, Shandong University, Jinan, Shandong 250012, China

**Keywords:** Major depressive disorder, Chronic unpredictable mild stress, PPP4R3A, Synthesis of synaptic proteins, mTORC1 signaling

## Abstract

Chronic stress is one of the main risk factors for the onset of major depressive disorder. Chronic unpredictable mild stress results in reduced expression of synaptic proteins and depression-like behaviors in rodent models. However, the upstream molecule that senses the demand for synaptic proteins and initiates their synthesis under chronic stress remains unknown. In this study, chronic unpredictable mild stress reduced the expression of PPP4R3A in the prefrontal cortex and hippocampus in mice. Selective knockout of *Ppp4r3a* in the cortex and hippocampus mimicked the depression- and anxiety-like behavioral effects of chronic stress in mice. Notably, *Ppp4r3a* deficiency led to downregulated mTORC1 signaling, which resulted in reduced synthesis of synaptic proteins and impaired synaptic functions. By contrast, overexpression of *Ppp4r3a* in the cortex and hippocampus protected against behavioral and synaptic deficits induced by chronic stress in a PPP4R3A–mTORC1-dependent manner. Rapamycin treatment of *Ppp4r3a*-overexpressing neurons blocked the regulatory effect of *Ppp4r3a* on the synthesis of synaptic proteins by directly inhibiting mTORC1. Overall, our results reveal a regulatory role of *Ppp4r3a* in driving synaptic protein synthesis in chronic stress.

## Abstract (precis)

**Summary:** Chronic unpredictable mild stress, a putative model of depression, reduced PPP4R3A expression in mice. PPP4R3A deficiency in mice leads to depression-like behaviors by inhibiting mTORC1-mediated synthesis of synaptic proteins.

## INTRODUCTION

Major depressive disorder (MDD) is one of the most common and disabling mental disorders, affecting an estimated 350 million people globally and causing enormous personal suffering and societal economic burdens (Whiteford et al., 2013; Otte et al., 2016). MDD can be a lethal illness because of the increased risk of suicide, cardiac disease and cerebrovascular disease (Walker et al., 2015). Current pharmacotherapies [selective serotonin reuptake inhibitors (SSRIs)] for MDD require a prolonged time (weeks, if not months) to produce clinical improvement (Rush et al., 2006). The high incidence of partial or no response, along with the delayed onset of antidepressant effects, leaves many patients with inadequate treatment (Rush et al., 2006). A single subanesthetic dose of ketamine can induce rapid (within hours) and robust (up to 7 days) antidepressant responses in patients (Berman et al., 2000). However, the risks of neurotoxicity, cystitis, dissociative side effects and abuse of ketamine make it difficult for this treatment to gain widespread acceptance for clinical use (Bonaventura et al., 2021). Thus, identifying genetic changes related to depressive disorders and finding novel antidepressant targets are prospective directions for MDD therapy.

Consistent evidence from studies on the etiology and treatment of MDD are suggestive of the central role of homeostatic control of synaptic connections (Duman and Aghajanian, 2012; Duman et al., 2016). Previous postmortem studies demonstrated decreased expression of synapse-related genes, reduced number of synapses and dendritic complexity, associated with neuronal atrophy and decreased brain volume in MDD patients (Drevets, 2000; MacQueen et al., 2008; Kang et al., 2012). Chronic unpredictable mild stress (CUMS) in rodents is a putative model of depression (Willner et al., 1987; Kallarackal et al., 2013). Studies on rodents have revealed decreased levels of synaptic proteins, reduced synaptic density, neuronal atrophy and cell loss in depression-like mice under chronic stress (Radley et al., 2006; Kang et al., 2012). Recent reports suggest that the rapid antidepressant actions of ketamine depend on fast induction of synaptogenesis, which can persist for a long time and can, therefore, reverse the synaptic deficits and anhedonia caused by chronic stress (Berman et al., 2000; Zarate et al., 2006). Regulation of synaptic protein synthesis is the cornerstone for homeostatic control of synaptic connections; it is effected by complex signaling molecules, including neurotransmitters, cytokines, growth and neurotrophic factors, energy and metabolic factors, sex steroids and the hypothalamic–pituitary–adrenal axis (Duman and Aghajanian, 2012). The mammalian target of rapamycin complex 1 (mTORC1) cascade is the convergent downstream pathway that directly drives the translation of synaptic proteins (Sonenberg and Hinnebusch, 2009). However, the upstream mediator involved in sensing of complex signaling changes and in modulating the synthesis of synaptic proteins under chronic stress remains unclear.

Protein phosphatase 4 (PP4), which belongs to the PP2A-type phosphatases (including PP2AC, PP4C and PP6C), is a protein complex composed of a catalytic subunit PP4C and regulatory subunits (Gingras et al., 2005). PP2A was reported to participate in various neurodegenerative diseases, and, in particular, inhibition of PP2A activity was found in sporadic Alzheimer's disease (AD), focal cerebral ischemia and traumatic brain injury (Koh, 2011; Sontag and Sontag, 2014; Shultz et al., 2015). Notably, PP2A inhibition attenuated depression-like symptoms in a learned helplessness model of depression in mice (Lecca et al., 2016). Protein phosphatase 4 regulatory subunit 3A (*PPP4R3A*), also known as suppressor of MEK null 1 (*SMEK1*), is a regulatory subunit of PP4 enzyme. In *Dictyostelium*, *smek* is necessary for defects in polarity, chemotaxis speed and directionality in *mek1*-null and *erk1*-null cells, which accounts for the name *smek* (Mendoza et al., 2005). PPP4R3A has been reported to play an important role in the nervous system. PPP4R3A deficiency prevented neural stem cells from differentiating, leading to neurogenesis deficits during mouse cortical development (Lyu et al., 2013; Chang et al., 2017; Moon et al., 2017). A protective *PPP4R3A* variant, rs2273647-T, was identified in AD patients that helps to slow the onset and progression of AD pathology via reduced glucose uptake by the brain (Christopher et al., 2017). In addition, *PPP4R3A* plays an important role in the maintenance of embryonic stem cell pluripotency, hepatic gluconeogenesis, glucose metabolism, tumor suppression, tumor angiogenesis, microRNA biogenesis and transcription initiation (Yoon et al., 2010; Lyu et al., 2011; Byun et al., 2012; Dong et al., 2012; Ma et al., 2014; Su et al., 2017; Sen et al., 2020; Wang et al., 2021). Some neurogenesis-related or neurodegenerative disorder-related genes have been proven to be involved in depressive disorders (Choi et al., 2018; Ntim et al., 2020). However, the role of *PPP4R3A* in emotional regulation is poorly understood.

In the present study, we noticed reduced expression of PPP4R3A in CUMS-exposed depression-like mice. To determine whether *Ppp4r3a* is involved in depression and is related to the synthesis of synaptic proteins, we analyzed the behavioral effects and synaptic basis in *Ppp4r3a*-knockout and -overexpressing (OE) mice. Importantly, we endeavored to reveal the role of *Ppp4r3a* in chronic stress-induced depression and to find the bridge by which *Ppp4r3a* regulates the synthesis of synaptic proteins. In summary, we concentrated on the regulatory mechanism of synaptic synthesis in depression and investigated the molecular basis of depression to find possible novel therapeutic targets.

## RESULTS

### CUMS treatment reduces PPP4R3A expression in the prefrontal cortex (PFC) and hippocampus

To study the role of PPP4R3A in the central nervous system, we analyzed its expression profile in the brains of wild-type mice. Specific cohorts of mice used for analysis are shown in Fig. S1. In adult wild-type (WT) mice, PPP4R3A was widely expressed in regions of brain, including the olfactory bulb, PFC, hippocampus, striatum, substantia nigra, hypothalamus, medulla oblongata and cerebellum (Fig. S2A). Next, we detected PPP4R3A expression in tissues from WT mice at postnatal day (P)0, P3, P5, P7, P10, P14, P21 and P28, and observed that expression in the hippocampus progressively decreased during development and became stable after P14 (Fig. S2B). Nuclear localization of PPP4R3A was confirmed by co-immunostaining of PPP4R3A with NeuN (also known as RBFOX3), GFAP and IBA1 in brain slices of adult WT mice, revealing PPP4R3A immunoreactivity in neurons, astrocytes and microglia, respectively (Fig. S2C).

**Fig. 1.**
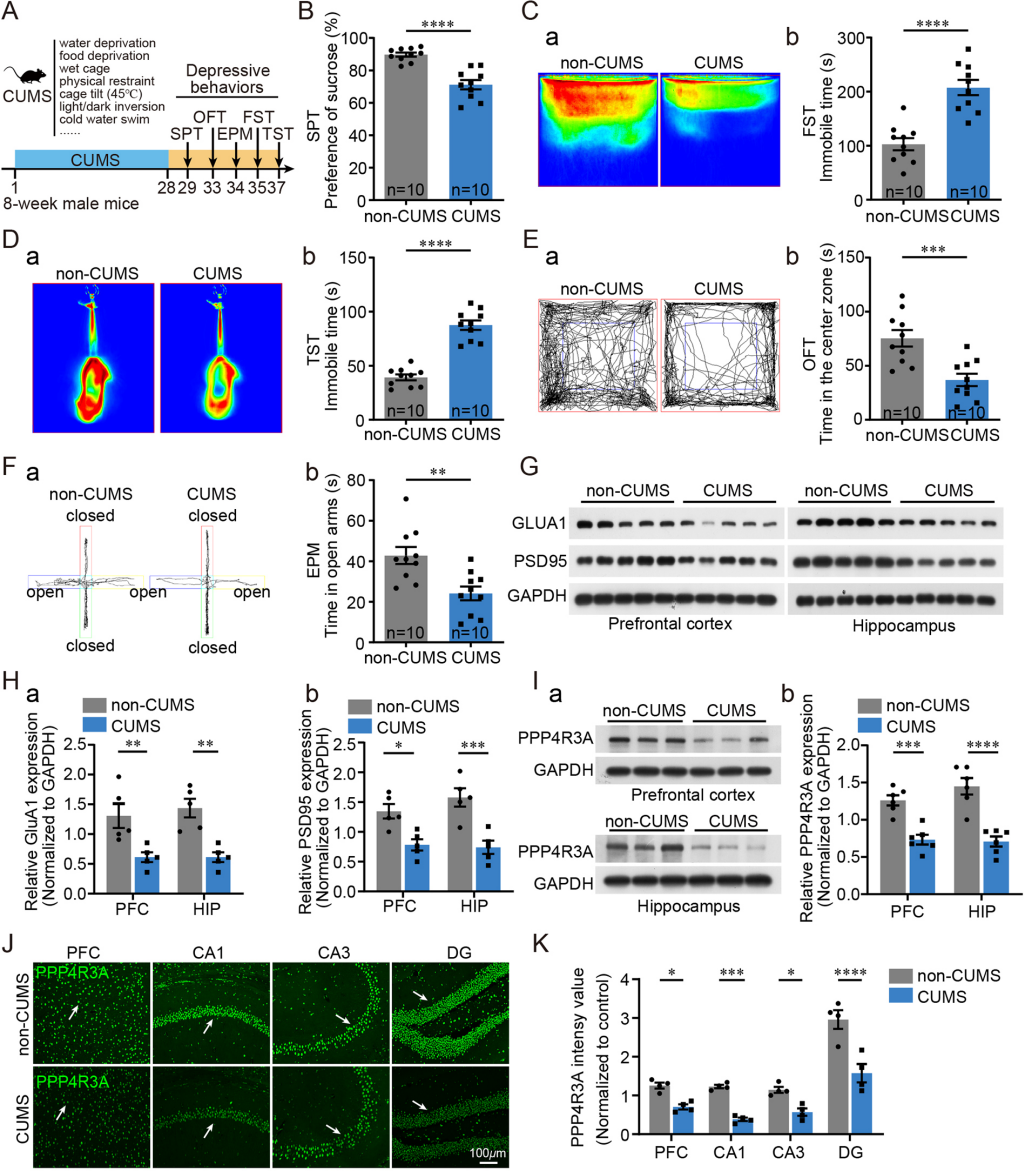
**Chronic unpredictable mild stress (CUMS) treatment reduces PPP4R3A expression in the prefrontal cortex (PFC) and hippocampus.** (A) Outline of the CUMS experimental design. Mice were exposed to CUMS for 28 days, followed by assessment of depression-like behaviors. EPM, elevated plus maze; FST, forced swim test; OFT, open field test; SPT, sucrose preference test; TST, tail suspension test. (B) Results of SPT. CUMS-exposed mice showed a decreased preference for sucrose compared with non-CUMS mice [unpaired two-tailed Student's *t*-test, *t=*5.909, degrees of freedom (d.f.)*=*18, ****P=0.0001; non-CUMS *n*=10, CUMS *n*=10]. (C) Results of FST. (a) Representative movement heatmap showing the less active state of CUMS-induced mice. The activity states of high, low and immobile for mice are represented in red, yellow and green, respectively. (b) Immobile time was increased in CUMS-induced mice (unpaired Student's *t*-test, *t=*5.812, d.f.*=*18, ****P=0.0001). (D) Results of TST. (a) Representative movement heatmap showing the less active state of CUMS-induced mice. (b) Immobile time was increased in CUMS-induced mice (unpaired Student's *t*-test, *t=*9.288, d.f.*=*18, ****P=0.0001). (E) Results of OFT. (a) Representative movement paths showing a decreased tendency of CUMS-exposed mice to move in the center zone. (b) Time spent in the center zone was decreased in CUMS-induced mice (unpaired Student's *t*-test, *t=*4.037, d.f.*=*18, ***P=0.0008). (F) Results of EPM test. (a) Representative movement paths, showing reduced interest in exploring the open arms in CUMS-exposed mice. (b) Time spent in the open arms was decreased in CUMS-induced mice (unpaired Student's *t*-test, *t=*3.480, d.f.*=*18, **P=0.0027). (G) Decreased expression of GluA1 and PSD95 in the PFC and hippocampus in CUMS-exposed mice (*n*=5). (H) Quantification of protein band intensity in Fig. 1G (*n*=5). (a) GluA1 (two-way ANOVA, main effect of CUMS, *F*_4,90_=0.2674, *P=*0.8982; interaction, *F*_4,90_=0.2674, *P=*0.8982; Bonferroni post hoc test, **P<0.01). (b) PSD95 (two-way ANOVA, main effect of CUMS, *F*_1,16_=32.56, *P*<0.0001; interaction, *F*_1,16_=1.243, *P=*0.2813; Bonferroni post hoc test, *P<0.05, ***P<0.001). (I) Decreased expression of PPP4R3A in the PFC and hippocampus of CUMS-exposed mice. (a) Representative western blotting results (3:3). (b) Quantification of protein band intensity in western blot analysis based on six independent results for the PFC and hippocampus (two-way ANOVA, main effect of CUMS, *F*_1,20_=62.16, *P*<0.0001; interaction, *F*_1,20_=1.770, *P=*0.1984; Bonferroni post hoc test, ***P<0.001, ****P<0.0001; non-CUMS *n*=6, CUMS *n*=6). (J) Representative immunostaining images showing decreased expression of PPP4R3A (white arrows) in the PFC and hippocampus in CUMS-induced mice. (K) Quantification of the luminosity of PPP4R3A-immunoreactive signals shown in J (two-way ANOVA, main effect of CUMS, *F*_1,24_=78.84, *P*<0.0001; interaction, *F*_3,20_=4.194, *P=*0.0161; Bonferroni post hoc test, *P<0.05, ***P<0.001, ****P<0.0001; non-CUMS *n*=4, CUMS *n*=4).

In rodent models of CUMS, exposure to unpredictable stress for several weeks results in anhedonia and synapse defects, which are core symptoms of depression (Willner et al., 1987; Kallarackal et al., 2013). We exposed WT male C57BL/6 mice to 28 days of CUMS and validated the effects on depression using behavioral tests, including the sucrose preference test (SPT), forced swim test (FST), tail suspension test (TST), open field test (OFT) and elevated plus maze (EPM) test (Fig. 1A). To ensure the successful depression modeling of CUMS, all mice were subjected to the behavioral tasks. Detailed experimental procedures for behavioral tests are provided in the Supplementary Materials and Methods. As expected, CUMS-exposed mice showed reduced preference for sucrose in the SPT trials, which assessed anhedonia of mice (Fig. 1B). In the FST and TST trials, assessing behavioral despair in an escapable and struggling environment (indicating depression-like behavior), CUMS-exposed mice showed increased immobile time (Fig. 1C,D). In OFT trials, CUMS-exposed mice showed reduced time spent in the center zone, indicating anxiety-like behavior (Fig. 1E). In EPM trials, CUMS-exposed mice showed decreased exploratory time spent in the open arms, also a clue of anxiety-like behavior (Fig. 1F). These behavioral changes confirmed successful depression modeling in mice after CUMS exposure. Although carryover effects might emerge in mice after a series of behavioral tasks, we exposed CUMS and non-CUMS mice to identical procedures, thus minimizing the potential confounding effects for subsequent analysis.

Next, we isolated the PFC and hippocampus from CUMS-exposed and control mice to examine the expression levels of synaptic proteins. The results suggested reduced levels of GluA1 (also known as GRIA1) and PSD95 (also known as DLG4), which are needed for the formation and maturation of synapses, in the PFC and hippocampus (Fig. 1G,H). Importantly, we found that the PPP4R3A level was significantly reduced in the PFC and hippocampus in CUMS-exposed mice using western blot and immunostaining analyses (Fig. 1I-K). Thus, our data demonstrate reduced expression of PPP4R3A in CUMS-exposed mice, suggesting its possible involvement in the development of depression-like behaviors in mice.

### *Ppp4r3a* deficiency in the cortex and hippocampus leads to depression- and anxiety-like behaviors

**Fig. 2.**
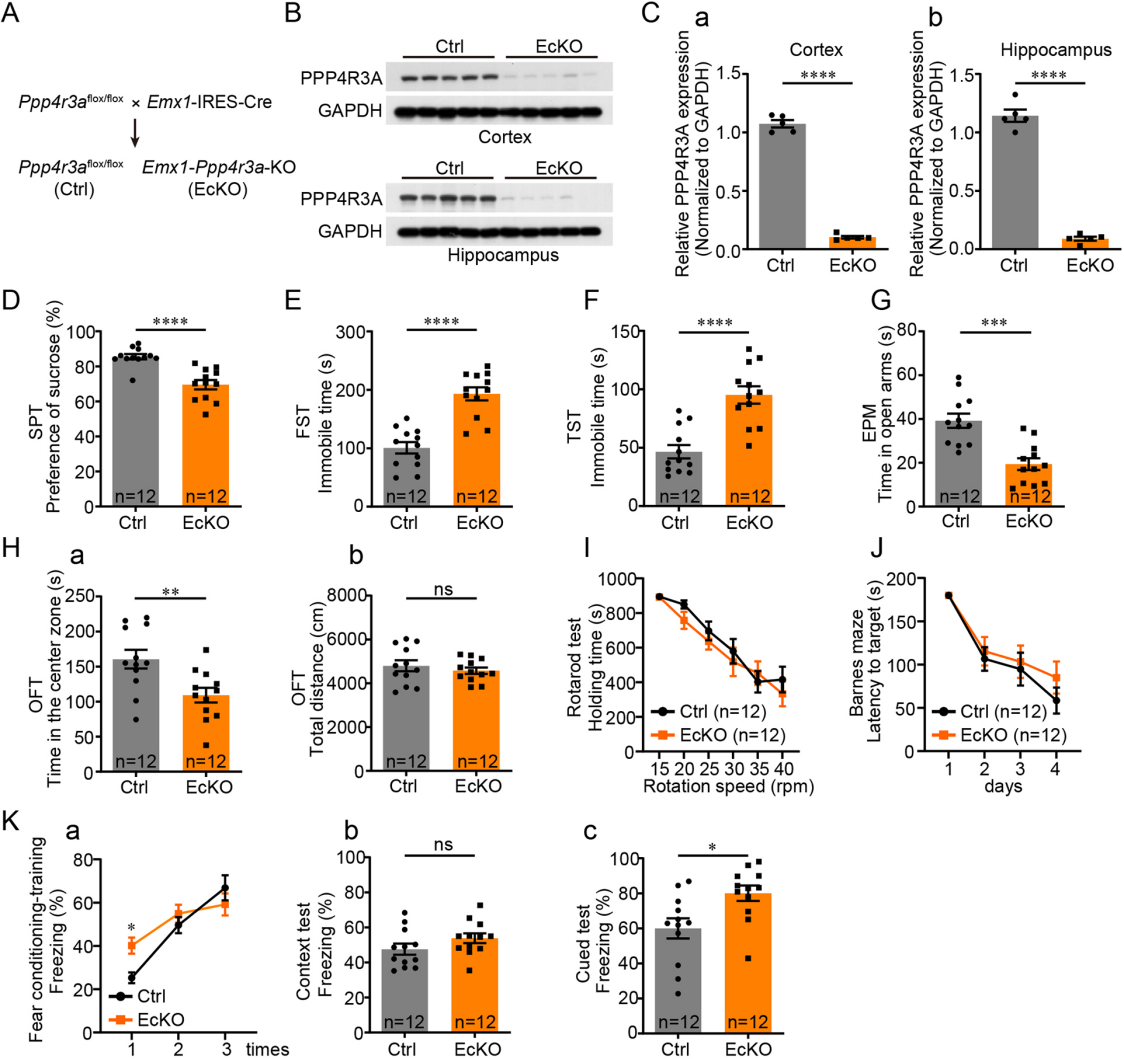
***Ppp4r3a* deficiency in the cortex and hippocampus leads to depression- and anxiety-like behaviors.** (A) Strategy for the generation of the conditionally cortical and hippocampal *Ppp4r3a* knockout [*Emx1* conditionally knockout (EcKO)] mice by crossbreeding *Ppp4r3a*^flox/flox^ mice with *Emx1*-IRES-Cre mice. (B) Western blot results showing effective knockout of PPP4R3A in the cortex and hippocampus in EcKO mice. (C) Quantification of band intensity in the western blots shown in Fig. 2B. (a) Cortex [unpaired Student's *t*-test, *t=*28.83, d.f.*=*8, ****P<0.0001; control (Ctrl) *n*=5, EcKO *n*=5]. (b) Hippocampus (unpaired Student's *t*-test, d.f.*=*8, *t=*19.15, ****P<0.0001). (D) Results of SPT. EcKO mice showed a decreased preference for sucrose compared with Ctrl mice (unpaired Student's *t*-test, *t=*5.227, d.f.*=*22, ****P<0.0001; Ctrl *n*=12, EcKO *n*=12). (E) Results of FST. Immobile time was increased in EcKO mice compared with that in Ctrl mice (unpaired Student's *t*-test, d.f.*=*22, *t=*6.221, ****P<0.0001). (F) Results of TST. Immobile time was increased in EcKO mice compared with that in Ctrl mice (unpaired Student's *t*-test, d.f.*=*22, *t=*5.171, *P*<0.0001). (G) Results of EPM test. Time spent in the open arms was decreased in EcKO mice compared with Ctrl mice (unpaired Student's *t*-test, d.f.*=*22, *t=*4.727, ***P=0.0001). (H) Results of OFT. (a) Decreased time spent in the center zone was observed in EcKO mice compared with Ctrl mice (unpaired Student's *t*-test, d.f.*=*22, *t=*3.037, **P=0.006). (b) No difference in moving distance was observed in EcKO mice compared with Ctrl mice (unpaired Student's *t*-test, d.f.*=*22, *t=*0.7787, *P=*0.4445). (I) Results of rotarod test. Normal motor endurance in holding the rotating rod was observed in EcKO mice compared with Ctrl mice (two-way ANOVA, main effect of genotype, *F*_1,132_=1.592, *P=*0.2093). (J) Results of Barnes maze test. No difference was observed in the time spent finding the target in EcKO and Ctrl mice (two-way ANOVA, main effect of genotype, *F*_1,88_=1.109, *P=*0.2953). (K) Results of fear conditioning tests. (a) Training period. Increased freezing time in responding to the first foot shock was observed in EcKO mice compared with Ctrl mice (two-way ANOVA, main effect of genotype, *F*_1.66_=1.532, *P=*0.2202; Bonferroni post hoc test, *P<0.05). No difference in the freezing time was observed after three training sessions, as baseline response to shock. (b) No difference in the freezing time in context-induced fear memory testing was observed in EcKO mice compared with Ctrl mice (unpaired Student's *t*-test, d.f.*=*22, *t=*1.484, *P=*0.1519). (c) Increased freezing time in cue-induced fear memory testing was observed in EcKO mice compared with Ctrl mice (unpaired Student's *t*-test, d.f.*=*22, *t=*2.772, *P=0.0111). ns, not significant.

To explore the effects of *Ppp4r3a* in the cortex and hippocampus, we specifically deleted the gene in the cortex and hippocampus by crossbreeding *Emx1*-IRES-Cre mice with *Ppp4r3a*^flox/flox^ mice (Fig. 2A; Fig. S3A). Effective knockdown of PPP4R3A in the cortex and hippocampus of mice was evident in western blot analyses (Fig. 2B,C). The *Emx1* conditionally knockout (EcKO) mice had normal birth rate, survival rate and body weight compared to those of WT mice. The whole brain size and weight of EcKO mice showed no apparent differences from those of the WT mice (Fig. S3B,C). Additionally, no appreciable differences in cortical thickness or hippocampal size or number of neurons were observed by Nissl staining (Fig. S3D).

Next, we performed a global analysis of depression-like, anxiety-like, locomotion, spatial learning and memory behaviors of mice. Given the basal motional endurance differences between female and male mice in behavioral tests, we obtained results for female and male EcKO mice, and similar trends were shown for both sexes in all the behavioral tests. Adult 8-week-old EcKO mice spontaneously showed diminished sucrose preference during the SPT trials, and increased immobility time in the FST and TST trials, indicating depression-like behavior (Fig. 2D-F). In addition, EcKO mice showed reduced interest in exploring the open arms in EPM trials and decreased time spent in the center zone in OFT, indicating anxiety-like behavior (Fig. 2G,H). Normal locomotion activity of EcKO mice was evident from the similar movement distance in OFT trials compared with that of control mice and normal motor endurance in rotarod tests (Fig. 2H,I). By Barnes maze tests with adult 8-week-old EcKO and control mice, normal spatial learning and memory skills were observed in finding targets in EcKO mice (Fig. 2J). Fear conditioning is a form of learning through which individuals learn the relationships between aversive stimuli and other conditions, and is implicated in the pathogenesis of anxiety. In particular, anxious individuals are hypothesized to show stronger fear learning that is more resistant to extinction (Uys et al., 2003; Indovina et al., 2011). Thus, we performed context- and cue-associated fear conditioning tests on adult 8-week-old EcKO and control mice to further assess their anxiety-like traits. During the training process, EcKO mice showed increased reactivity to the first electric shock and displayed similar levels of freezing after three repeats of the training. During the testing process, EcKO mice showed similar memories for context-induced fear and strengthened memories for cue-induced fear (in new compartment) (Fig. 2K). Thus, the increased reactivity for sudden fear stimuli and stronger learning memory for fear suggested an anxiety-like trait in the EcKO mice. In addition, hot plate tests were performed to assess the sensory difficulties of mice. The results showed no differences in foot withdrawal latency in adult 8-week-old EcKO mice compared with control mice, indicating normal sensory functions (Fig. S4G). In summary, our results demonstrate that loss of PPP4R3A in the cortex and hippocampus led to elevated anxiety- and depression-like behaviors in mice.

### *Ppp4r3a* deficiency in the hippocampus leads to impaired synaptic functions and disrupted synaptic synthesis

**Fig. 3.**
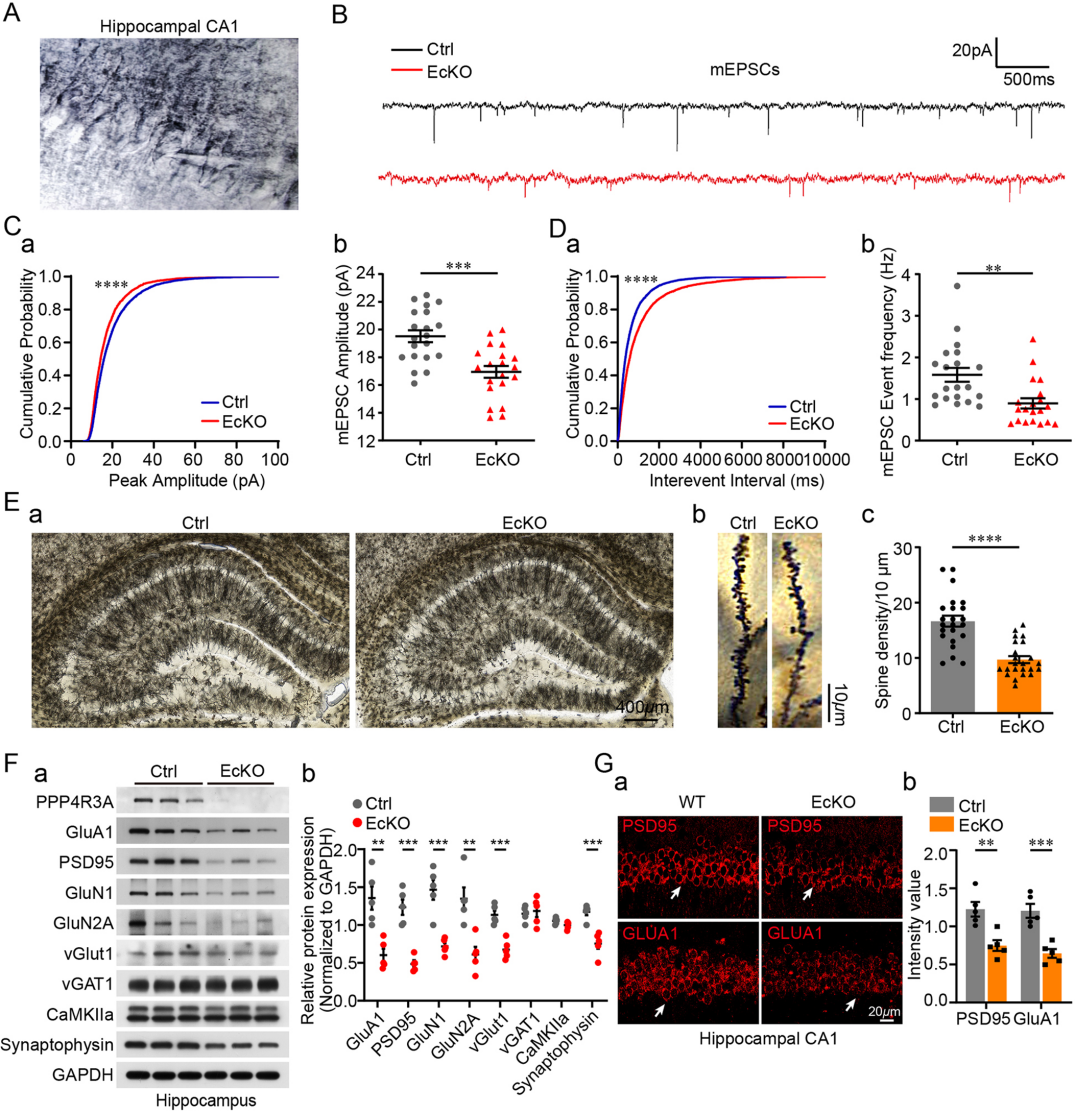
***Ppp4r3a* deficiency in the hippocampus leads to impaired synaptic functions and disrupted synaptic synthesis.** (A) A representative image showing the position at which miniature excitatory postsynaptic currents (mEPSCs) were recorded from hippocampal CA1 pyramidal neurons. (B) Representative and average traces of mEPSCs recorded from hippocampal CA1 pyramidal neurons in WT (black) and EcKO mice (red). (C) mEPSC amplitude. (a) The cumulative probability of mEPSC amplitude was smaller in EcKO mice than in littermate Ctrl mice (Kolmogorov–Smirnov test, ****P<0.0001). (b) Decreased average mEPSC amplitude in EcKO mice (unpaired Student's *t*-test, *t=*4.263, d.f.*=*38, ***P=0.0002, *n*=20 neurons from six mice per group). (D) mEPSC interevent interval. (a) The cumulative probability of the mEPSC interevent interval was higher in EcKO mice than in Ctrl mice (Kolmogorov–Smirnov test, ****P<0.0001). (b) Decreased mEPSC event frequency in EcKO mice compared with Ctrl mice (unpaired Student's *t*-test, *t=*2.680, d.f.*=*38, **P=0.0117, *n*=20 neurons from six mice per group). (E) Golgi staining of 8-week-old EcKO and littermate Ctrl mice. (a) Representative Golgi staining from hippocampal CA1 regions. (b) Representative projection images of dendritic spines from hippocampal neurons. (c) Quantification of dendritic spine density showing decreased spine density in EcKO mice compared with Ctrl mice (unpaired Student's *t*-test, *t=*3.32, d.f.=46, ****P<0.0001, *n*=23 neurons from three mice per group). (F) Expression levels of synapse-related proteins in the hippocampus of Ctrl and EcKO mice. Decreased expression levels of glutamate receptors (GluA1, GluN1, and GluN2A), PSD95, vGlut1 and synaptophysin were observed in EcKO mice compared with Ctrl mice. (a) Representative bands observed in western blot analysis (3:3). (b) Quantification of the intensity of protein bands (unpaired Student's *t*-test, d.f.=8, **P<0.01, ***P<0.001). (G) Immunostaining images of the hippocampus showing decreased expression of GluA1 and PSD95 in EcKO mice compared with Ctrl mice. (a) Representative immunostaining images (white arrows indicate immunoreactive signals). (b) Quantification of the luminosity of GluA1 or PSD95 in immunostaining images (two-way ANOVA, main effect of genotype, *F*_1,16_=41.71, *P*<0.0001; interaction, *F*_1,16_=0.2498, *P=*0.6240; Bonferroni post hoc test, **P<0.01, ***P<0.001; Ctrl *n*=5, EcKO *n*=5).

To explore the synaptic basis in the hippocampus underlying aberrant depression- and anxiety-like behaviors in EcKO mice, we recorded the miniature excitatory postsynaptic currents (mEPSCs) in the hippocampal CA1 pyramidal neurons (Fig. 3A,B). The results demonstrated reduced amplitude of mEPSCs in EcKO mice, suggesting decreased expression of postsynaptic receptors (Fig. 3C). The frequency of mEPSCs in EcKO mice was reduced, indicating decreased release of the presynaptic excitatory neurotransmitter, glutamate (Fig. 3D). Next, we investigated whether the impairment of excitatory synaptic transmission was associated with alterations in the number of synapses and expression of glutamate receptors. Golgi staining showed reduced dendritic spine density in the hippocampus in EcKO mice (Fig. 3E). Notably, decreased expression of several NMDA receptor and AMPA receptor (AMPAR) subunits [GluA1, GluN1 (also known as GRIN1) and GluN2A (also known as GRIN2A)] and several excitatory synapse-related proteins [vGlut1 (also known as SLC17A7), synaptophysin and PSD95] were observed in KO mice (Fig. 3F). No changes in expression of markers of inhibitory synapses (vGAT1, officially known as SLC32A1) and excitatory neurons (Camk2a) were observed (Fig. 3F). The decreased GluA1 and PSD95 levels in EcKO mice were further confirmed by immunostaining of the hippocampus sections (Fig. 3G). In summary, *Ppp4r3a* deficiency leads to synaptic deficits in the hippocampus, characterized by reduced expression of synapse-related proteins, decreased dendritic density and impairment of excitatory glutamate transmission.

### *Ppp4r3a* drives hippocampal synaptic protein synthesis by activating the mTORC1 cascade

**Fig. 4.**
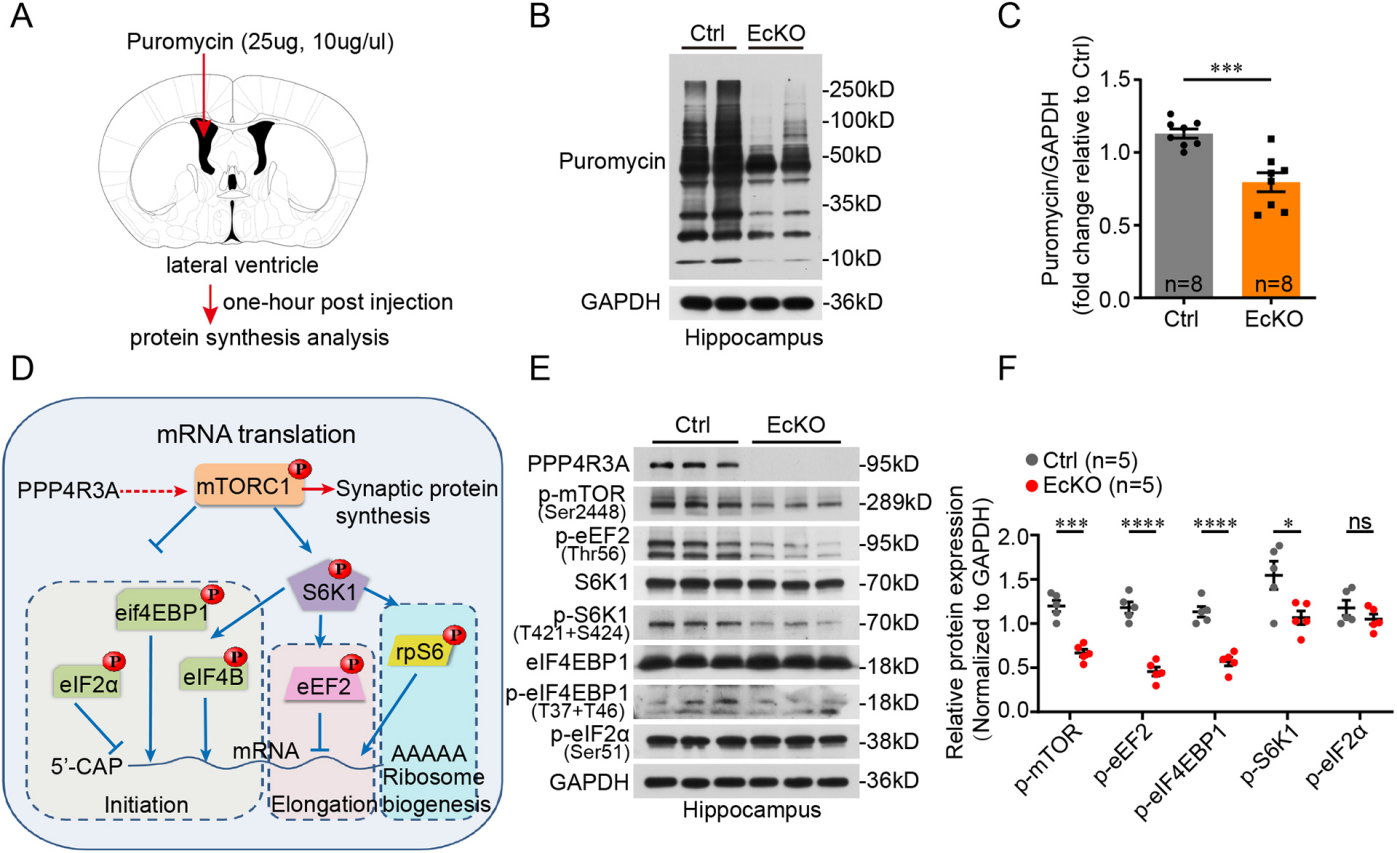
***Ppp4r3a* drives hippocampal synaptic protein synthesis by activating the mTORC1 cascade.** (A) Schematic design of the surface sensing of translation (SUnSET) experiment *in vivo*. Puromycin was injected into the lateral ventricle of EcKO and littermate Ctrl mice, and the puromycin-labeled proteins were examined 1 h after the injection. (B) Representative blots showing decreased levels of puromycin-labeled proteins in the hippocampus of EcKO mice. (C) Quantification of puromycin-labeled proteins [normalized to the control cell line, unpaired Student's *t*-test, *t*=4.611, d.f.=14, ***P<0.001; Ctrl (*n*=8), EcKO (*n*=8)]. (D) Schematic representation of the roles of *Ppp4r3a* in regulating mRNA translation in an mTORC1-dependent (through phosphorylation of mTOR and its substrates) or mTORC1-indepedent (through phosphorylation of eIF2ɑ) manner. (E) Immunoblot analysis of representative markers of mTORC1 signaling in the hippocampus of Ctrl and EcKO mice. Decreased expression of phosphorylated (p)-mTOR, p-eEF2, p-S6K1 and p-eIF4EBP1 in EcKO mice. (F) Quantification of the intensity of protein bands in E [unpaired Student's *t*-test, d.f.=8, *P<0.05, ***P<0.001, ****P<0.0001; Ctrl (*n*=5), EcKO (*n*=5)]. ns, not significant.

The alteration of synaptic protein synthesis by regulating cap-dependent mRNA translation is a crucial pathway for rapid antidepressant response (Sonenberg and Hinnebusch, 2009; Aguilar-Valles et al., 2021). Thus, we examined the global protein synthesis in the brain of WT and EcKO mice using a technique called surface sensing of translation (SUnSET) (Schmidt et al., 2009). We injected puromycin into the lateral ventricle of mice to label newly synthesized polypeptides *in vivo* (Fig. 4A). The levels of puromycin-labeled proteins in the hippocampus were significantly decreased in EcKO mice (Fig. 4B,C). Thus, *Ppp4r3a* deficiency led to lower protein synthesis, consistent with the decrease in GluA1 and PSD95 levels in the hippocampus of EcKO mice. To further verify that the diminished expression of synaptic proteins was because of reduced protein synthesis and not excessive protein degradation, we constructed *PPP4R3A*-OE and *PPP4R3A*-deficient SH-SY5Y cell lines to analyze the relationship of PPP4R3A with GluA1 and PSD95. Notably, upon treatment with MG132 (an inhibitor of proteasome-mediated protein degradation), the positive regulatory effect of *PPP4R3A* on GluA1 and PSD95 remained unchanged (Fig. S5A). Upon treatment with cycloheximide (an inhibitor of protein synthesis), protein degradation remained unchanged (Fig. S5B). Therefore, *Ppp4r3a* positively drives synaptic protein synthesis by triggering mRNA translation in the hippocampus.

Activation of mTORC1 is critical for the initiation of cap-dependent mRNA translation via the phosphorylation of two key effectors, eIF4E-binding proteins (4E-BPs) and p70S6 kinase 1 (S6K1) (Hoeffer and Klann, 2010) (Fig. 4D). Diminished mTORC1 signaling in the hippocampus of *Ppp4r3a*-knockout mice was indicated by reduced levels of phosphorylated mTOR, S6K1, eIF4EBP1 and eEF2 (Fig. 4E,F). The phosphorylation level of eIF2α, an inhibitor of mRNA translation initiation through an mTORC1-independent pathway, was not changed (Fig. 4E,F). In addition, the regulatory effect of *PPP4R3A* on mTORC1 was further verified in *PPP4R3A*-OE or sh*PPP4R3A* SH-SY5Y cells (Fig. S5C). ERK (also known as MAPK) and PKB/AKT signaling was also detected in the *PPP4R3A*-OE SH-SY5Y cells, which are known to be critical for the activation of mTORC1 and need further analysis in the future (Fig. S5D). In summary, PPP4R3A drives the synthesis of synaptic proteins by activating the mTORC1 cascade.

### *Ppp4r3a* overexpression in mice protects against CUMS-induced effects by increasing mTORC1 activity

**Fig. 5.**
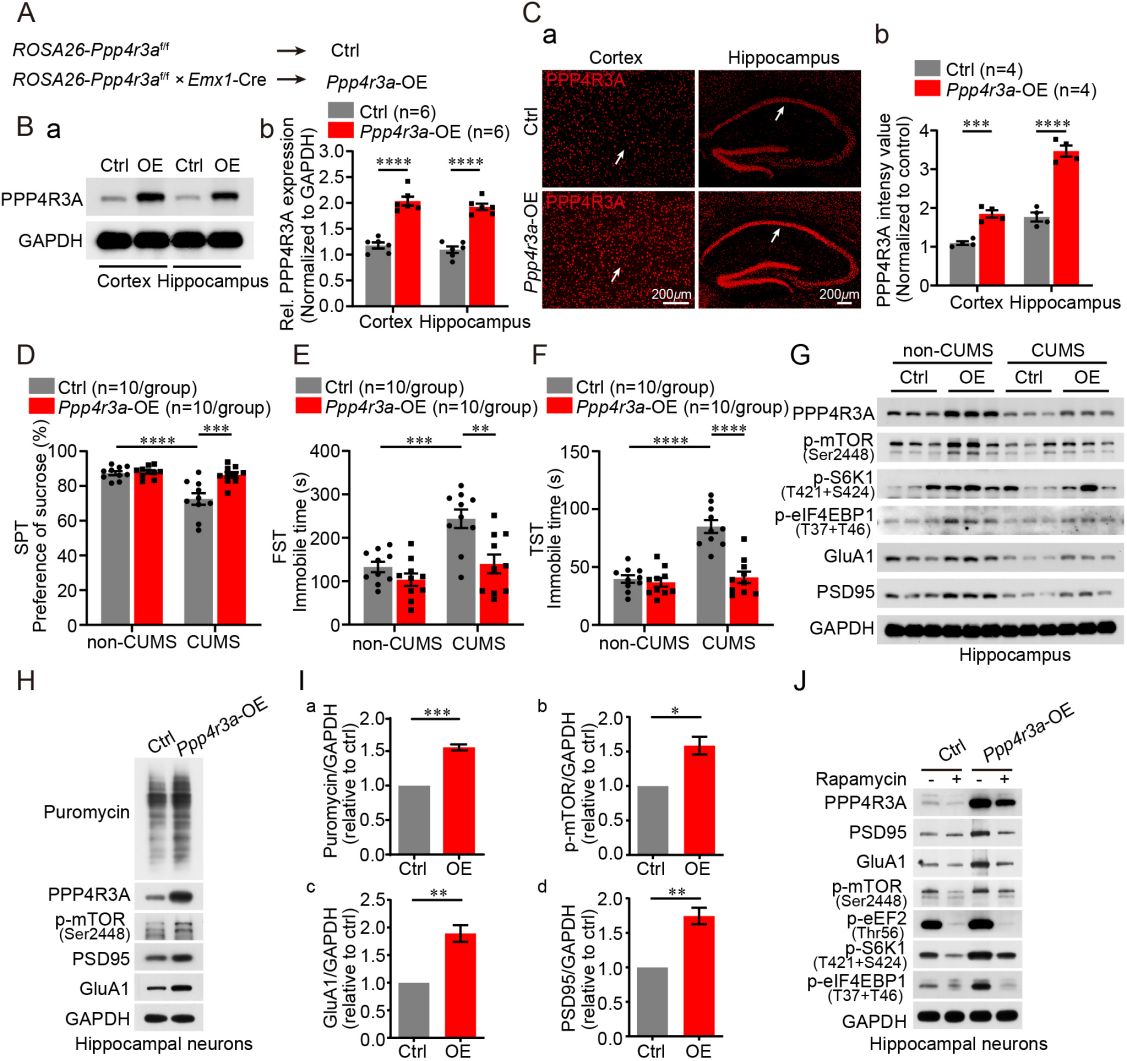
***Ppp4r3a* overexpression in mice protects against CUMS-induced effects by increasing mTORC1 activity.** (A) Generation of cortex- and hippocampus-specific *Ppp4r3a*-overexpressing (OE) mice by crossbreeding *ROSA26*-*Ppp4r3a*^flox/flox^ mice with *Emx1*-IRES-Cre mice. (B) Immunoblot results showing effective overexpression of PPP4R3A in the cortex and hippocampus. (a) Representative western blot showing the protein band. (b) Quantification of band intensity from six independent experiments (two-way ANOVA, main effect of genotype, *F*_1,20_=154.4, *P*<0.0001; interaction, *F*_1,20_=10.04937, *P=*0.8264; Bonferroni post hoc test, ****P<0.0001; Ctrl *n*=6, *Ppp4r3a*-OE *n*=6). (C) Immunostaining images showing effective overexpression of PPP4R3A in the cortex and hippocampus of *Ppp4r3a*-OE mice. (a) Representative immunostaining images. (b) Quantification of PPP4R3A luminosity in immunostaining images (two-way ANOVA, main effect of genotype, *F*_1,12_=132.4, *P*<0.0001; interaction, *F*_1,12_=19.79, *P=*0.0008; Bonferroni post hoc test, ***P<0.001, ****P<0.0001; Ctrl *n*=4, *Ppp4r3a*-OE *n*=4). (D) Results of SPT. *Ppp4r3a*-OE mice were protected against the decrease in preference for sucrose induced by CUMS compared with that in Ctrl mice (two-way ANOVA, main effect of genotype, *F*_1,36_=13.08, *P=*0.0009; main effect of CUMS, *F*_1,36_=17.06, *P=*0.0002; interaction, *F*_1,36_=11.02, *P=*0.0021; Bonferroni post hoc test, ***P<0.001, ****P<0.0001; Ctrl *n*=10, *Ppp4r3a*-OE *n*=10). (E) Results of FST. *Ppp4r3a*-OE mice were protected against the increase in immobile time induced by CUMS compared with that in Ctrl mice (two-way ANOVA, main effect of genotype, *F*_1,36_=13.96, *P=*0.0006; main effect of CUMS, *F*_1,36_=17, *P=*0.0002; interaction, *F*_1,36_=4.391, *P=*0.0432; Bonferroni post hoc test, **P<0.01, ***P<0.001). (F) Results of TST. *Ppp4r3a*-OE mice were protected against the increase in immobile time induced by CUMS compared with that in Ctrl mice (two-way ANOVA, main effect of genotype, *F*_1,36_=27.63, *P*<0.0001; main effect of CUMS, *F*_1,36_=31.09, *P*<0.0001; interaction, *F*_1,36_=21.23, *P*<0.0001; Bonferroni post hoc test, ****P<0.0001). (G) Results of immunoblot analysis, suggesting that protection of *Ppp4r3a*-OE mice against the decrease in the levels of synaptic proteins (GluA1 and PSD95) induced by CUMS is independent of mTORC1 signaling. (H) Compared to neurons from control mice, increased levels of puromycin-labeled proteins, p-mTOR, GluA1 and PSD95 were observed in *Ppp4r3a*-OE hippocampal neurons from *Ppp4r3a*-OE mice at embryonic day 18.5. (I) Quantification of puromycin-labeled proteins [(a) unpaired Student's *t*-test, d.f.=4, *t*=4.723, *P*=0.0092], p-mTOR [(b) unpaired Student's *t*-test, d.f.=4, *t*=4.473, *P*=0.0102], GluA1 [(c) unpaired Student's *t*-test, d.f.=4, *t*=5.280, *P*=0.0062] and PSD95 [(d) unpaired Student's *t*-test, d.f.=4, *t*=6.244, *P*=0.0034]. We ran the experiment in triplicate, and the expression of control neurons was normalized as 1 for every repeated experiment. (J) Immunoblot analysis showing that rapamycin treatment of *Ppp4r3a*-OE hippocampal neurons blocked the positive regulatory effect of *Ppp4r3a* on the synthesis of synaptic proteins (GluA1 and PSD95) by directly inhibiting the mTORC1 signaling.

To investigate whether *Ppp4r3a* overexpression could rescue depression-like behavioral defects and synaptic deficits in CUMS-induced mice, we overexpressed *Ppp4r3a* in the cortex and hippocampus of mice by crossbreeding *ROSA26*-*Ppp4r3a*^flox/flox^ mice with *Emx1*-IRES-Cre mice (Fig. 5A; Fig. S2E). Effective overexpression of PPP4R3A in the cortex and hippocampus of *Ppp4r3a*-OE mice was observed using western blot and immunostaining analyses (Fig. 5B,C). After 28 days of CUMS, *Ppp4r3a*-OE mice showed resistance to CUMS-induced depression-like behaviors compared with control mice, as confirmed by normal sucrose preference in the SPT and normal immobile time in the FST or TST (Fig. 5D-F). Next, we focused on the reduced mTORC1 signaling and synaptic proteins in CUMS-induced mice. As expected, *Ppp4r3a*-OE mice exhibited attenuated CUMS-induced downregulation of mTORC1 signaling and disrupted synaptic protein synthesis in the hippocampus, consistent with the protective effects in behavioral tests (Fig. 5G; Fig. S6A). Thus, replenishing PPP4R3A in the cortex and hippocampus was sufficient for correcting defective depression-like behaviors and disrupted synthesis of synaptic proteins caused by CUMS.

Using cultured primary hippocampal neurons from the *Ppp4r3a*-OE mice, we further validated the positive regulatory effect of *Ppp4r3a* on synaptic protein synthesis via mTORC1-mediated mRNA translation (Fig. 5H,I). Rapamycin (an inhibitor of mTORC1) treatment of *Ppp4r3a*-OE hippocampal neurons blocked the positive regulation of synaptic protein synthesis by *Ppp4r3a* through the direct blocking of mTORC1 (Fig. 5J; Fig. S6B). In addition, rapamycin treatment of *PPP4R3A*-OE SH-SY5Y cells also restored increased synthesis of GluA1 and PSD95 by inhibiting mTORC1 directly (Fig. S6C). Together, our findings suggest a significant role of PPP4R3A in driving synaptic protein synthesis and in resisting depression-like behaviors in chronic stress by activating the mTORC1 cascade.

## DISCUSSION

Chronic stress is a potential trigger for MDD. In our study, in CUMS-exposed depressive mice, diminished expression of PPP4R3A was observed in the PFC and hippocampus, suggesting the involvement of *Ppp4r3a* in the development of depression. Furthermore, mice with *Ppp4r3a* deficiency in the cortex and hippocampus mimicked CUMS-induced depression- and anxiety-like behaviors. The overexpression of *Ppp4r3a* in the cortex and hippocampus of mice rescued CUMS-induced depression-like behaviors, indicating that *Ppp4r3a* is a protective factor under chronic stress. Together, these findings suggest prospects for *Ppp4r3a* in depression therapy.

The significant role of increased synaptic protein synthesis in antidepressant therapy has been previously noted. Many factors have been related to the disrupted expression of synapse-related proteins, which increases the susceptibility to depression. For identifying novel targets for antidepressant therapy, it is essential to clarify the regulatory mechanism of synaptic protein synthesis. The mTORC1 cascade is the known downstream convergence pathway crucial for synthesis of new proteins and synaptogenesis, and it regulates the initiation and elongation of mRNA translation (Sonenberg and Hinnebusch, 2009; Hoeffer and Klann, 2010). Previous postmortem studies in MDD patients indicated an association between deficits in mTOR-dependent mRNA translation and deficits in synaptic protein synthesis in the PFC (Feyissa et al., 2009; Jernigan et al., 2011). Preclinical and clinical studies have shown that the molecular basis for the rapid and robust antidepressant effects of ketamine is the acute activation of mTORC1, which leads to persistent synaptogenesis and behavioral relief (Li et al., 2010). Another study showed that 4E-BP1 and 4E-BP2, the key downstream effectors of mTORC1, are central to the antidepressant activity of ketamine (Aguilar-Valles et al., 2021). In addition, mTORC1 activation is the major effect of chronic treatment with conventional antidepressant drugs and SSRIs (Dagestad et al., 2006; Liu et al., 2015). Therefore, clarifying the role of an endogenous genetic inducer of the mTORC1 cascade in modulating synaptic protein synthesis is meaningful for antidepressant therapy.

Herein, we provide direct evidence that *Ppp4r3a* deficiency impairs synaptic protein synthesis and synaptogenesis and consequently results in depression-like behaviors by inhibiting the mTORC1 cascade. Examination of the synaptic basis in *Ppp4r3a*-deficient mice revealed the dysfunction of hippocampal synapses, including disrupted synthesis of synaptic proteins, reduced dendritic spine density and diminished excitatory glutamate synaptic transmission. *Ppp4r3a* deficiency in mice decreased mTORC1 signaling and reduced synaptic protein synthesis in the hippocampus, in accordance with impairment of hippocampal excitatory synaptic function and depression-like behaviors. The overexpression of *Ppp4r3a* in mice attenuated downregulation of mTORC1 and disrupted synaptic protein synthesis, thus leading to relief in depression-like behaviors. Rapamycin treatment of *Ppp4r3a-*OE hippocampal neurons blocked the positive regulatory effect of *Ppp4r3a* on synaptic protein synthesis by directly inhibiting mTORC1. Overall, our findings show that Ppp4r3a is a genetic inducer of mTORC1 and synaptic protein synthesis.

The activation of ERK and PKB/AKT is related to the activation of mTORC1 (Bockaert and Marin, 2015). Ketamine administration activates ERK, PKB/AKT and mTORC1 signaling in mice, and treatment with an ERK inhibitor (U0126), a PI3K inhibitor (LY294003) or rapamycin blocks ketamine-induced increases in mTORC1 phosphorylation and synaptogenesis (Li et al., 2010). Studies on the role of mTORC1 in the antidepressant effects of ketamine and its two enantiomers have shown that (S)-ketamine requires mTORC1, but (R)-ketamine requires ERK, activation (Yang et al., 2018). In *in vitro* experiments, we identified activation of PKB/AKT and ERK signaling in *PPP4R3A-*OE cells. The specific mechanism of triggering of mTORC1 signaling by PPP4R3A to produce rapid antidepressant responses needs to be further investigated in the future.

Intriguingly, we noticed the positive regulatory effect of PPP4R3A on GluA1 and PSD95, which are needed for the maturation of existing synapses and for the formation of new ones. GluA1, a very important subunit of AMPARs, induces the trafficking and integration of AMPARs (Zhang and Abdullah, 2013). Previous studies have shown that adult hippocampal long-term potentiation was absent in GluA1-deficient mice, but this synapse modification in CA1 was not required for spatial learning (Zamanillo et al., 1999; Mack et al., 2001). Our findings that *Ppp4r3a*-deficient mice demonstrate synapse dysfunction but display normal spatial learning memory further validates this viewpoint. Increased expression and phosphorylation of GluA1 are common therapeutic targets for the treatment of depression, schizophrenia, chronic drug addiction and AD (Eastwood et al., 1997; Duman et al., 2019; Qu et al., 2021). Moreover, increased GluA1 expression causes pain and is involved in epilepsy (Su et al., 2015; Joshi et al., 2018). PSD95, the principal scaffold protein in the postsynaptic density, regulates the anchorage, trafficking and localization of glutamate receptors, thus playing a role in MDD (Ehrlich and Malinow, 2004). Other studies have shown that the aberrant expression of PSD95 is associated with schizophrenia, AD, Huntington's disease, autism spectrum disorders and pain disorder (Arbuckle et al., 2010; de Bartolomeis et al., 2014; Savioz et al., 2014; Zhang et al., 2014). Thus, the positive regulatory effect of PPP4R3A on GluA1 and PSD95 indicates that PPP4R3A might play a more extensive role in psychiatric or neurodegenerative disorders and needs to be explored further.

In summary, PPP4R3A is a positive genetic regulator of synaptic protein synthesis, which activates the mTORC1 cascade, thereby playing an essential role in hippocampal synaptogenesis, maintenance of dendritic spine density and excitatory glutamate synaptic transmission. PPP4R3A is involved in chronic stress-induced depression and is a promising biomarker and therapeutic target for depressive disorder.

## MATERIALS AND METHODS

### Animals

All animal experiments were conducted according to standard research protocols approved by the Animal Care and Use Committee, School of Basic Medical Sciences, Shandong University. Animals were group housed and maintained on a 12 h light/dark cycle at a constant temperature of 22°C, with free access to food and water. To analyze the expression pattern of PPP4R3A in mice, WT C57BL6/J mice were used for western blotting and immunostaining. To demonstrate the role of PPP4R3A in CUMS-induced depression, adult litter/age-matched male C57BL/6J mice (8 weeks old) were used for CUMS modeling and subsequent behavioral tests, immunostaining and western blot analysis. Then, to evaluate the impact of PPP4R3A deficiency *in vivo*, *Ppp4r3a* knockout mice were used for behavioral analysis, mEPSCs trials, Golgi staining and protein synthesis analysis. To further verify the role of PPP4R3A under CUMS pressure, *Ppp4r3a*-OE mice were used for behavioral analysis and protein synthesis analysis under CUMS modeling. Detailed cohorts of mice used for specific experimental analysis are shown in Fig. S1. All behavioral studies were conducted by an experimenter blinded to the genotypes of the mice.

### CUMS

We used a modified CUMS procedure described by Jaggar et al. (2017) and Nasca et al. (2013). C57BL/6 WT male mice (8 weeks old) were divided into control (*n*=10) and CUMS (*n*=15) groups. Mice in the CUMS group were maintained in individual cages and continuously received the following stress for 28 days: (1) 12 h water deprivation and 2 h exposure to an empty bottle; (2) 12 h food deprivation; (3) 12 h exposure to a wet cage (21°C water, filled 2 cm above the cage bottom); (4) 2 h of physical restraint in stainless steel retainers; (5) 12 h of cage tilt (45°); (6) 24 h of light/dark inversion; (7) 5 min of forced swimming at 4-8°C; (8) cold exposure at 4°C for 45 min; (9) overcrowding without bedding for 3 h (five mice in a 1 l beaker, 10 cm×13.5 cm); (10) cage exchange for 3 h (empty cage of other mice). The detailed procedure for 28 days is presented in Table S1. Mice were subjected to one or two stressors per day with an adequate interval for recovery. The above stressors were carried out randomly every week, and the behavioral tests were conducted 24 h after the last stressor to assess the depressive-like behaviors. The timeline of the behavioral tests is shown in Fig. 1A.

### Generation of *Ppp4r3a* knockout mice

*Ppp4r3a* selectively knockout mice, EcKO mice, were generated following a standard Cre-LoxP recombination strategy. *Ppp4r3a*^flox/flox^ mice were generated by Cyagen Biosciences Inc. (Guangzhou, China). The targeting vector included a Rox-flanked Neo cassette, two homology arms and two loxP loci. The linearized vector was delivered to embryonic stem cells through electroporation, followed by drug selection, PCR screening and Southern blot verification. Positive F0 chimera mice were crossed with WT mice to generate F1 heterozygous *Ppp4r3a*^flox/+^ mice. *Emx1*-IRES-Cre (The Jackson Laboratory, Stock No. 005628) mice have an Emx1 promoter driving Cre recombinase expression in ∼88% of the neurons of the neocortex and hippocampus. Thus, we crossbred *Ppp4r3a*^flox/flox^ mice with *Exm1*-IRES-Cre mice to selectively knock out *Ppp4r3a* in the cortex and hippocampus.

### Generation of *Ppp4r3a*-OE mice

*Ppp4r3a*-OE mice (C57BL/6) were generated using the standard Cre-LoxP system. *ROSA26-Ppp4r3a*^flox/flox^ mice were generated by Biocytogen Corporation (Beijing, China) using the Biocytogen Extreme Genome Editing system. The targeting vector included a CAG promoter, a loxP-Stop-loxP transcriptional stop element and a *Ppp4r3a* CDS-HA tag-WPRE-polyA element. The single-guide RNA (sgRNA) was designed near the ROSA26 insertion site. Cas9/sgRNA and the targeting vector were microinjected into the fertilized eggs of mice to obtain chimeric F0 mice. F1 mice were obtained by crossing positive F0 mice with WT mice. *ROSA26-Ppp4r3a*^flox/flox^ mice were mated with *Emx1*-IRES-Cre mice to overexpress *Ppp4r3a* in the cortex and hippocampus of mice.

### Behavioral analysis

The SPT is the gold standard test to assess depression-like behavior in rodents by assessing anhedonia (core symptom of depression). The FST and TST assess despair behavior by measuring immobile time in an inescapable environment, allowing a simple assessment of potential antidepressant activity. The OFT and EPM test are routinely used to assess anxiety-like behaviors in rodents, and the OFT is also used to provide a qualitative and quantitative measurement of exploratory and locomotor activity. The Barnes maze is a dry-land maze that is commonly used for measuring spatial learning and memory in rodents. The rotarod test is used for assessing general locomotion. Fear conditioning is a form of learning that is implicated in the pathogenesis of anxiety disorders, thus stronger fear learning and memory in mice indicate increased degree of anxiety. The hot plate test is used for assessing basal sensory–motor disturbance in mice. The behavioral tasks of mice were performed in the order represented in the flowchart in Fig. S1. Detailed experimental procedures are provided in the Supplementary Materials and Methods.

### mEPSCs

Postnatal 8-week-old EcKO mouse and their control littermates were prepared for mEPSC tests. In brief, hippocampal slices were prepared by coronal sectioning (300 μm thick) using a vibrating slicer (Leica, VT 1000 S, Germany) in oxygenated ice-cold cutting solution. The prepared slices were immediately transferred to artificial cerebrospinal fluid (ACSF) at 32°C and incubated for 30 min. Whole-cell patch-clamp recordings were performed in hippocampal CA1 cells. For recording, slices were continuously perfused with ACSF at a flow rate of 6 ml/min with a temperature at 31±1°C. Cells were visualized with a microscope (BX51-WI, Olympus, Tokyo, Japan). After adding 1 μM tetrodotoxin (TTX) and 10 μM bicuculline in the ACSF, mEPSCs were recorded at a holding clamping voltage at −70 mV. The experimental data were recorded using a MultiClamp 700B amplifier and Clampfit 10.6 software (both Molecular Devices, San Jose, CA, USA) and analyzed by Prism 8.0.2 (GraphPad Software, San Diego, CA, USA) to calculate the current amplitude and discharge frequency, reflecting the presynaptic glutamate release and expression of postsynaptic receptors.

### Immunofluorescence staining

Each mouse was dissected immediately after transcardial perfusion with 4% paraformaldehyde (PFA). The brain was isolated and post-fixed in 4% PFA overnight. Fixed tissues were serially sectioned (4 μm) after paraffin embedding. The sections were dewaxed with gradient xylene and anhydrous ethanol, antigen repaired with high temperature, blocked with 10% goat or donkey serum, and immunostained with specific antibodies. After immunostaining, the sections were panoramically scanned with a scanning microscope and were analyzed with CaseViewer 2.0 software (3D HISTECH, Budapest, Hungary). Some representative images were obtained through a fluorescence microscope (Olympus).

### Cell culture

SH-SY5Y cells were obtained from our laboratory storage and were cultured in Dulbecco's modified Eagle medium containing 1% glutamate, 10% fetal bovine serum and maintained at 37°C with 5% CO_2_ in a humidified atmosphere.

### Statistical analysis

Data analysis was performed using GraphPad Prism 8.0. Data are displayed as mean±s.e.m. Statistical differences between two groups (non-CUMS versus CUMS or control versus EcKO or control versus *PPP4R3A*-OE) were generally analyzed using an unpaired Student's *t-*test. *P*<0.05 was considered statistically significant. Two-way ANOVA followed by Bonferroni post hoc test was applied for analyzing behavioral tests of *Ppp4r3a*-OE mice and control mice with or without CUMS and was applied for analyzing social behaviors. The Kolmogorov–Smirnov test was used to analyze the cumulative probability of mEPSC amplitude and interevent intervals.

## Supplementary Material

Supplementary information

## References

[DMM049374C1] Aguilar-Valles, A., De Gregorio, D., Matta-Camacho, E., Eslamizade, M. J., Khlaifia, A., Skaleka, A., Lopez-Canul, M., Torres-Berrio, A., Bermudez, S., Rurak, G. M. et al. (2021). Antidepressant actions of ketamine engage cell-specific translation via eIF4E. *Nature* 590, 315-319. 10.1038/s41586-020-03047-0 33328636

[DMM049374C2] Arbuckle, M. I., Komiyama, N. H., Delaney, A., Coba, M., Garry, E. M., Rosie, R., Allchorne, A. J., Forsyth, L. H., Bence, M., Carlisle, H. J. et al. (2010). The SH3 domain of postsynaptic density 95 mediates inflammatory pain through phosphatidylinositol-3-kinase recruitment. *EMBO Rep.* 11, 473-478. 10.1038/embor.2010.63 20467438 PMC2892321

[DMM049374C3] Berman, R. M., Cappiello, A., Anand, A., Oren, D. A., Heninger, G. R., Charney, D. S. and Krystal, J. H. (2000). Antidepressant effects of ketamine in depressed patients. *Biol. Psychiatry* 47, 351-354. 10.1016/s0006-3223(99)00230-9 10686270

[DMM049374C4] Bockaert, J. and Marin, P. (2015). mTOR in brain physiology and pathologies. *Physiol. Rev.* 95, 1157-1187. 10.1152/physrev.00038.2014 26269525

[DMM049374C5] Bonaventura, J., Lam, S., Carlton, M., Boehm, M. A., Gomez, J. L., Solís, O., Sánchez-Soto, M., Morris, P. J., Fredriksson, I., Thomas, C. J. et al. (2021). Pharmacological and behavioral divergence of ketamine enantiomers: implications for abuse liability. *Mol. Psychiatry*. 26, 6704-6722. 10.1038/s41380-021-01093-2 33859356 PMC8517038

[DMM049374C6] Byun, H. J., Kim, B. R., Yoo, R., Park, S. Y. and Rho, S. B. (2012). sMEK1 enhances gemcitabine anti-cancer activity through inhibition of phosphorylation of Akt/mTOR. *Apoptosis* 17, 1095-1103. 10.1007/s10495-012-0751-0 22903553

[DMM049374C7] Chang, W.-H., Choi, S. H., Moon, B.-S., Cai, M., Lyu, J., Bai, J., Gao, F., Hajjali, I., Zhao, Z., Campbell, D. B. et al. (2017). Smek1/2 is a nuclear chaperone and cofactor for cleaved Wnt receptor Ryk, regulating cortical neurogenesis. *Proc. Natl. Acad. Sci. USA* 114, E10717-E10725. 10.1073/pnas.1715772114 29180410 PMC5740651

[DMM049374C8] Choi, J. E., Lee, J. J., Kang, W., Kim, H. J., Cho, J. H., Han, P. L. and Lee, K. J. (2018). Proteomic analysis of hippocampus in a mouse model of depression reveals neuroprotective function of ubiquitin C-terminal hydrolase L1 (UCH-L1) via stress-induced cysteine oxidative modifications. *Mol. Cell. Proteomics* 17, 1803-1823. 10.1074/mcp.RA118.000835 29959188 PMC6126396

[DMM049374C9] Christopher, L., Napolioni, V., Khan, R. R., Han, S. S. and Greicius, M. D. and Alzheimer's Disease Neuroimaging Initiative. (2017). A variant in PPP4R3A protects against alzheimer-related metabolic decline. *Ann. Neurol.* 82, 900-911. 10.1002/ana.25094 29130521 PMC5752155

[DMM049374C10] Dagestad, G., Kuipers, S. D., Messaoudi, E. and Bramham, C. R. (2006). Chronic fluoxetine induces region-specific changes in translation factor eIF4E and eEF2 activity in the rat brain. *Eur. J. Neurosci.* 23, 2814-2818. 10.1111/j.1460-9568.2006.04817.x 16817885

[DMM049374C11] de Bartolomeis, A., Latte, G., Tomasetti, C. and Iasevoli, F. (2014). Glutamatergic postsynaptic density protein dysfunctions in synaptic plasticity and dendritic spines morphology: relevance to schizophrenia and other behavioral disorders pathophysiology, and implications for novel therapeutic approaches. *Mol. Neurobiol.* 49, 484-511. 10.1007/s12035-013-8534-3 23999870

[DMM049374C12] Dong, S. M., Byun, H. J., Kim, B. R., Lee, S. H., Trink, B. and Rho, S. B. (2012). Tumor suppressor BLU enhances pro-apoptotic activity of sMEK1 through physical interaction. *Cell. Signal.* 24, 1208-1214. 10.1016/j.cellsig.2012.02.002 22349239

[DMM049374C13] Drevets, W. C. (2000). Functional anatomical abnormalities in limbic and prefrontal cortical structures in major depression. *Prog. Brain Res.* 126, 413-431. 10.1016/S0079-6123(00)26027-5 11105660

[DMM049374C14] Duman, R. S. and Aghajanian, G. K. (2012). Synaptic dysfunction in depression: potential therapeutic targets. *Science* 338, 68-72. 10.1126/science.1222939 23042884 PMC4424898

[DMM049374C15] Duman, R. S., Aghajanian, G. K., Sanacora, G. and Krystal, J. H. (2016). Synaptic plasticity and depression: new insights from stress and rapid-acting antidepressants. *Nat. Med.* 22, 238-249. 10.1038/nm.4050 26937618 PMC5405628

[DMM049374C16] Duman, R. S., Shinohara, R., Fogaça, M. V. and Hare, B. (2019). Neurobiology of rapid-acting antidepressants: convergent effects on GluA1-synaptic function. *Mol. Psychiatry* 24, 1816-1832. 10.1038/s41380-019-0400-x 30894661 PMC6754322

[DMM049374C17] Eastwood, S. L., Kerwin, R. W. and Harrison, P. J. (1997). Immunoautoradiographic evidence for a loss of alpha-amino-3-hydroxy-5-methyl-4-isoxazole propionate-preferring non-N-methyl-D-aspartate glutamate receptors within the medial temporal lobe in schizophrenia. *Biol. Psychiatry* 41, 636-643. 10.1016/S0006-3223(96)00220-X 9066986

[DMM049374C18] Ehrlich, I. and Malinow, R. (2004). Postsynaptic density 95 controls AMPA receptor incorporation during long-term potentiation and experience-driven synaptic plasticity. *J. Neurosci.* 24, 916-927. 10.1523/JNEUROSCI.4733-03.2004 14749436 PMC6729816

[DMM049374C19] Feyissa, A. M., Chandran, A., Stockmeier, C. A. and Karolewicz, B. (2009). Reduced levels of NR2A and NR2B subunits of NMDA receptor and PSD-95 in the prefrontal cortex in major depression. *Prog. Neuropsychopharmacol. Biol. Psychiatry* 33, 70-75. 10.1016/j.pnpbp.2008.10.005 18992785 PMC2655629

[DMM049374C20] Gingras, A. C., Caballero, M., Zarske, M., Sanchez, A., Hazbun, T. R., Fields, S., Sonenberg, N., Hafen, E., Raught, B. and Aebersold, R. (2005). A novel, evolutionarily conserved protein phosphatase complex involved in cisplatin sensitivity. *Mol. Cell. Proteomics* 4, 1725-1740. 10.1074/mcp.M500231-MCP200 16085932

[DMM049374C21] Hoeffer, C. A. and Klann, E. (2010). mTOR signaling: at the crossroads of plasticity, memory and disease. *Trends Neurosci.* 33, 67-75. 10.1016/j.tins.2009.11.003 19963289 PMC2821969

[DMM049374C22] Indovina, I., Robbins, T. W., Núñez-Elizalde, A. O., Dunn, B. D. and Bishop, S. J. (2011). Fear-conditioning mechanisms associated with trait vulnerability to anxiety in humans. *Neuron* 69, 563-571. 10.1016/j.neuron.2010.12.034 21315265 PMC3047792

[DMM049374C23] Jaggar, M., Weisstaub, N., Gingrich, J. A. and Vaidya, V. A. (2017). 5-HT 2A receptor deficiency alters the metabolic and transcriptional, but not the behavioral, consequences of chronic unpredictable stress. *Neurobiol. Stress* 7, 89-102. 10.1016/j.ynstr.2017.06.001 28626787 PMC5470573

[DMM049374C24] Jernigan, C. S., Goswami, D. B., Austin, M. C., Iyo, A. H., Chandran, A., Stockmeier, C. A. and Karolewicz, B. (2011). The mTOR signaling pathway in the prefrontal cortex is compromised in major depressive disorder. *Prog. Neuropsychopharmacol. Biol. Psychiatry* 35, 1774-1779. 10.1016/j.pnpbp.2011.05.010 21635931 PMC3154612

[DMM049374C25] Joshi, S., Sun, H., Rajasekaran, K., Williamson, J., Perez-Reyes, E. and Kapur, J. (2018). A novel therapeutic approach for treatment of catamenial epilepsy. *Neurobiol. Dis.* 111, 127-137. 10.1016/j.nbd.2017.12.009 29274741 PMC5803337

[DMM049374C26] Kallarackal, A. J., Kvarta, M. D., Cammarata, E., Jaberi, L., Cai, X., Bailey, A. M. and Thompson, S. M. (2013). Chronic stress induces a selective decrease in AMPA receptor-mediated synaptic excitation at hippocampal temporoammonic-CA1 synapses. *J. Neurosci.* 33, 15669-15674. 10.1523/JNEUROSCI.2588-13.2013 24089474 PMC3787493

[DMM049374C27] Kang, H. J., Voleti, B., Hajszan, T., Rajkowska, G., Stockmeier, C. A., Licznerski, P., Lepack, A., Majik, M. S., Jeong, L. S., Banasr, M. et al. (2012). Decreased expression of synapse-related genes and loss of synapses in major depressive disorder. *Nat. Med.* 18, 1413-1417. 10.1038/nm.2886 22885997 PMC3491115

[DMM049374C28] Koh, P. O. (2011). Focal cerebral ischemia reduces protein phosphatase 2A subunit B expression in brain tissue and HT22 cells. *Lab. Anim. Res.* 27, 73-76. 10.5625/lar.2011.27.1.73 21826165 PMC3145983

[DMM049374C29] Lecca, S., Pelosi, A., Tchenio, A., Moutkine, I., Lujan, R., Hervé, D. and Mameli, M. (2016). Rescue of GABAB and GIRK function in the lateral habenula by protein phosphatase 2A inhibition ameliorates depression-like phenotypes in mice. *Nat. Med.* 22, 254-261. 10.1038/nm.4037 26808347

[DMM049374C30] Li, N., Lee, B., Liu, R. J., Banasr, M., Dwyer, J. M., Iwata, M., Li, X. Y., Aghajanian, G. and Duman, R. S. (2010). mTOR-dependent synapse formation underlies the rapid antidepressant effects of NMDA antagonists. *Science* 329, 959-964. 10.1126/science.1190287 20724638 PMC3116441

[DMM049374C31] Liu, X. L., Luo, L., Mu, R. H., Liu, B. B., Geng, D., Liu, Q. and Yi, L. T. (2015). Fluoxetine regulates mTOR signalling in a region-dependent manner in depression-like mice. *Sci. Rep.* 5, 16024. 10.1038/srep16024 26522512 PMC4629199

[DMM049374C32] Lyu, J., Jho, E. H. and Lu, W. (2011). Smek promotes histone deacetylation to suppress transcription of Wnt target gene brachyury in pluripotent embryonic stem cells. *Cell Res.* 21, 911-921. 10.1038/cr.2011.47 21423269 PMC3203701

[DMM049374C33] Lyu, J., Kim, H.-R., Yamamoto, V., Choi, S. H., Wei, Z., Joo, C.-K. and Lu, W. (2013). Protein phosphatase 4 and Smek complex negatively regulate Par3 and promote neuronal differentiation of neural stem/progenitor cells. *Cell Rep* 5, 593-600. 10.1016/j.celrep.2013.09.034 24209749 PMC3855259

[DMM049374C34] Ma, H., Han, B. K., Guaderrama, M., Aslanian, A., Yates, J. R., 3rd, Hunter, T. and Wittenberg, C. (2014). Psy2 targets the PP4 family phosphatase Pph3 to dephosphorylate Mth1 and repress glucose transporter gene expression. *Mol. Cell. Biol.* 34, 452-463. 10.1128/MCB.00279-13 24277933 PMC3911506

[DMM049374C35] Mack, V., Burnashev, N., Kaiser, K. M., Rozov, A., Jensen, V., Hvalby, O., Seeburg, P. H., Sakmann, B. and Sprengel, R. (2001). Conditional restoration of hippocampal synaptic potentiation in Glur-A-deficient mice. *Science* 292, 2501-2504. 10.1126/science.1059365 11431570

[DMM049374C36] MacQueen, G. M., Yucel, K., Taylor, V. H., Macdonald, K. and Joffe, R. (2008). Posterior hippocampal volumes are associated with remission rates in patients with major depressive disorder. *Biol. Psychiatry* 64, 880-883. 10.1016/j.biopsych.2008.06.027 18722590

[DMM049374C37] Mendoza, M. C., Du, F., Iranfar, N., Tang, N., Ma, H., Loomis, W. F. and Firtel, R. A. (2005). Loss of SMEK, a novel, conserved protein, suppresses MEK1 null cell polarity, chemotaxis, and gene expression defects. *Mol. Cell. Biol.* 25, 7839-7853. 10.1128/MCB.25.17.7839-7853.2005 16107728 PMC1190274

[DMM049374C38] Moon, B. S., Yun, H. M., Chang, W. H., Steele, B. H., Cai, M., Choi, S. H. and Lu, W. (2017). Smek promotes corticogenesis through regulating Mbd3's stability and Mbd3/NuRD complex recruitment to genes associated with neurogenesis. *PLoS Biol.* 15, e2001220. 10.1371/journal.pbio.2001220 28467410 PMC5414985

[DMM049374C39] Nasca, C., Xenos, D., Barone, Y., Caruso, A., Scaccianoce, S., Matrisciano, F., Battaglia, G., Mathé, A. A., Pittaluga, A., Lionetto, L. et al. (2013). L-acetylcarnitine causes rapid antidepressant effects through the epigenetic induction of mGlu2 receptors. *Proc. Natl. Acad. Sci. USA* 110, 4804-4809. 10.1073/pnas.1216100110 23382250 PMC3607061

[DMM049374C40] Ntim, M., Li, Q.-F., Zhang, Y., Liu, X.-D., Li, N., Sun, H.-L., Zhang, X., Khan, B., Wang, B., Wu, Q. et al. (2020). TRIM32 deficiency impairs synaptic plasticity by excitatory-inhibitory imbalance via Notch pathway. *Cereb. Cortex* 30, 4617-4632. 10.1093/cercor/bhaa064 32219328

[DMM049374C41] Otte, C., Gold, S. M., Penninx, B. W., Pariante, C. M., Etkin, A., Fava, M., Mohr, D. C. and Schatzberg, A. F. (2016). Major depressive disorder. *Nat. Rev. Dis. Primers* 2, 16065. 10.1038/nrdp.2016.65 27629598

[DMM049374C42] Qu, W., Yuan, B., Liu, J., Liu, Q., Zhang, X., Cui, R., Yang, W. and Li, B. (2021). Emerging role of AMPA receptor subunit GluA1 in synaptic plasticity: implications for Alzheimer's disease. *Cell Prolif.* 54, e12959. 10.1111/cpr.12959 33188547 PMC7791177

[DMM049374C43] Radley, J. J., Rocher, A. B., Miller, M., Janssen, W. G., Liston, C., Hof, P. R., McEwen, B. S. and Morrison, J. H. (2006). Repeated stress induces dendritic spine loss in the rat medial prefrontal cortex. *Cereb. Cortex* 16, 313-320. 10.1093/cercor/bhi104 15901656

[DMM049374C44] Rush, A. J., Trivedi, , M. H., Wisniewski, , S. R., Nierenberg, A. A., Stewart, J. W., Warden, D., Niederehe, G., Thase, M. E., Lavori, P. W., Lebowitz, B. D. et al. (2006). Acute and longer-term outcomes in depressed outpatients requiring one or several treatment steps: a STAR*D report. *Am. J. Psychiatry* 163, 1905-1917. 10.1176/ajp.2006.163.11.1905 17074942

[DMM049374C45] Savioz, A., Leuba, G. and Vallet, P. G. (2014). A framework to understand the variations of PSD-95 expression in brain aging and in Alzheimer's disease. *Ageing Res. Rev.* 18, 86-94. 10.1016/j.arr.2014.09.004 25264360

[DMM049374C46] Schmidt, E. K., Clavarino, G., Ceppi, M. and Pierre, P. (2009). SUnSET, a nonradioactive method to monitor protein synthesis. *Nat. Methods* 6, 275-277. 10.1038/nmeth.1314 19305406

[DMM049374C47] Sen, I., Zhou, X., Chernobrovkin, A., Puerta-Cavanzo, N., Kanno, T., Salignon, J., Stoehr, A., Lin, X.-X., Baskaner, B., Brandenburg, S. et al. (2020). DAF-16/FOXO requires protein phosphatase 4 to initiate transcription of stress resistance and longevity promoting genes. *Nat. Commun.* 11, 138. 10.1038/s41467-019-13931-7 31919361 PMC6952425

[DMM049374C48] Shultz, S. R., Wright, D. K., Zheng, P., Stuchbery, R., Liu, S.-J., Sashindranath, M., Medcalf, R. L., Johnston, L. A., Hovens, C. M., Jones, N. C. et al. (2015). Sodium selenate reduces hyperphosphorylated tau and improves outcomes after traumatic brain injury. *Brain* 138, 1297-1313. 10.1093/brain/awv053 25771151 PMC5963409

[DMM049374C49] Sonenberg, N. and Hinnebusch, A. G. (2009). Regulation of translation initiation in eukaryotes: mechanisms and biological targets. *Cell* 136, 731-745. 10.1016/j.cell.2009.01.042 19239892 PMC3610329

[DMM049374C50] Sontag, J. M. and Sontag, E. (2014). Protein phosphatase 2A dysfunction in Alzheimer's disease. *Front. Mol. Neurosci.* 7, 16. 10.3389/fnmol.2014.00016 24653673 PMC3949405

[DMM049374C51] Su, C., D'Amour, J., Lee, M., Lin, H. Y., Manders, T., Xu, D., Eberle, S. E., Goffer, Y., Zou, A. H., Rahman, M. et al. (2015). Persistent pain alters AMPA receptor subunit levels in the nucleus accumbens. *Mol Brain* 8, 46. 10.1186/s13041-015-0140-z 26260133 PMC4531890

[DMM049374C52] Su, C., Li, Z., Cheng, J., Li, L., Zhong, S., Liu, L., Zheng, Y. and Zheng, B. (2017). The protein phosphatase 4 and SMEK1 complex dephosphorylates HYL1 to promote miRNA biogenesis by antagonizing the MAPK cascade in arabidopsis. *Dev. Cell* 41, 527-539.e5. 10.1016/j.devcel.2017.05.008 28586645

[DMM049374C53] Uys, J. D., Stein, D. J., Daniels, W. M. and Harvey, B. H. (2003). Animal models of anxiety disorders. *Curr. Psychiatry Rep.* 5, 274-281. 10.1007/s11920-003-0056-7 12857530

[DMM049374C54] Walker, E. R., McGee, R. E. and Druss, B. G. (2015). Mortality in mental disorders and global disease burden implications: a systematic review and meta-analysis. *JAMA Psychiatry* 72, 334-341. 10.1001/jamapsychiatry.2014.2502 25671328 PMC4461039

[DMM049374C55] Wang, Q., Wang, G., Niu, L., Zhao, S., Li, J., Zhang, Z., Jiang, H., Zhang, Q., Wang, H., Sun, P. et al. (2021). Exosomal MiR-1290 promotes angiogenesis of hepatocellular carcinoma via targeting SMEK1. *J Oncol.* 2021, 6617700. 10.1155/2021/6617700 33564307 PMC7864765

[DMM049374C56] Whiteford, H. A., Degenhardt, L., Rehm, J., Baxter, A. J., Ferrari, A. J., Erskine, H. E., Charlson, F. J., Norman, R. E., Flaxman, A. D., Johns, N. et al. (2013). Global burden of disease attributable to mental and substance use disorders: findings from the Global Burden of Disease Study 2010. *Lancet* 382, 1575-1586. 10.1016/S0140-6736(13)61611-6 23993280

[DMM049374C57] Willner, P., Towell, A., Sampson, D., Sophokleous, S. and Muscat, R. (1987). Reduction of sucrose preference by chronic unpredictable mild stress, and its restoration by a tricyclic antidepressant. *Psychopharmacology* 93, 358-364. 10.1007/BF00187257 3124165

[DMM049374C58] Yang, C., Ren, Q., Qu, Y., Zhang, J.-C., Ma, M., Dong, C. and Hashimoto, K. (2018). Mechanistic target of rapamycin-independent antidepressant effects of (R)-Ketamine in a social defeat stress model. *Biol. Psychiatry* 83, 18-28. 10.1016/j.biopsych.2017.05.016 28651788

[DMM049374C59] Yoon, Y.-S., Lee, M.-W., Ryu, D., Kim, J. H., Ma, H., Seo, W.-Y., Kim, Y.-N., Kim, S. S., Lee, C. H., Hunter, T. et al. (2010). Suppressor of MEK null (SMEK)/protein phosphatase 4 catalytic subunit (PP4C) is a key regulator of hepatic gluconeogenesis. *Proc. Natl. Acad. Sci. USA* 107, 17704-17709. 10.1073/pnas.1012665107 20876121 PMC2955085

[DMM049374C60] Zamanillo, D., Sprengel, R., Hvalby, O., Jensen, V., Burnashev, N., Rozov, A., Kaiser, K. M., Koster, H. J., Borchardt, T., Worley, P. et al. (1999). Importance of AMPA receptors for hippocampal synaptic plasticity but not for spatial learning. *Science* 284, 1805-1811. 10.1126/science.284.5421.1805 10364547

[DMM049374C61] Zarate, C. A., Jr, Singh, J. B., Carlson, P. J., Brutsche, N. E., Ameli, R., Luckenbaugh, D. A., Charney, D. S. and Manji, H. K. (2006). A randomized trial of an N-methyl-D-aspartate antagonist in treatment-resistant major depression. *Arch. Gen. Psychiatry* 63, 856-864. 10.1001/archpsyc.63.8.856 16894061

[DMM049374C62] Zhang, J. and Abdullah, J. M. (2013). The role of GluA1 in central nervous system disorders. *Rev. Neurosci.* 24, 499-505. 10.1515/revneuro-2013-0021 24077616

[DMM049374C63] Zhang, J., Saur, T., Duke, A. N., Grant, S. G., Platt, D. M., Rowlett, J. K., Isacson, O. and Yao, W. D. (2014). Motor impairments, striatal degeneration, and altered dopamine-glutamate interplay in mice lacking PSD-95. *J. Neurogenet.* 28, 98-111. 10.3109/01677063.2014.892486 24702501 PMC4406490

