# Supplementary material for: Ppp4r3a deficiency leads to depression-like behaviors in mice by modulating the synthesis of synaptic proteins
Source: Dis Model Mech. 2022 May 20;15(6):dmm049374. doi: 10.1242/dmm.049374 (PMC9150120; doi:10.1242/dmm.049374)
Supplement: Supplementary information [file dmm-15-049374-s1.pdf]

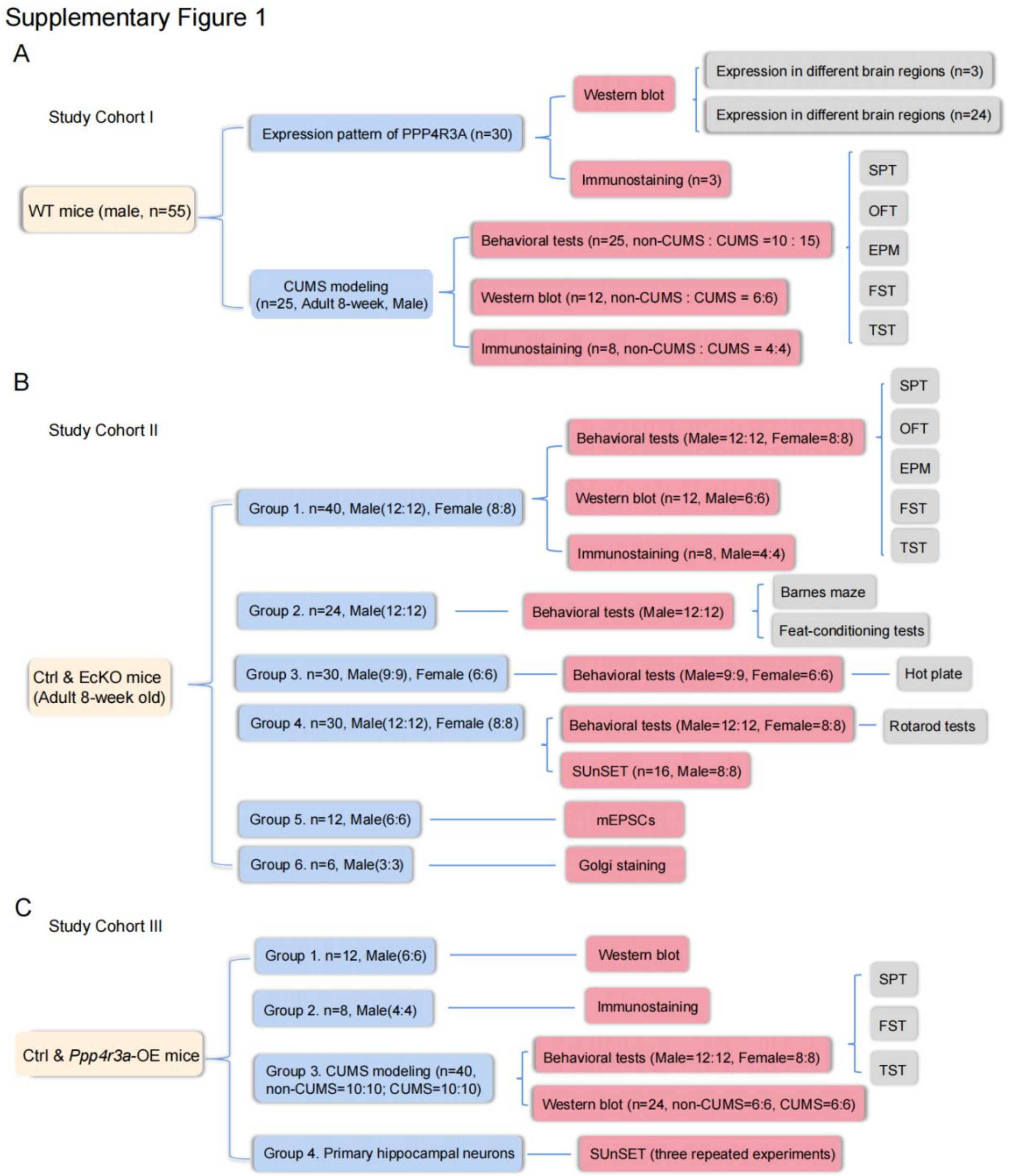

Fig. S1. The cohorts of mice used for the research.

(A) The detailed cohorts of wild-type mice used for analyzing the expression pattern of PPP4R3A and CUMS modeling.

(B) The detailed cohorts of EcKO and control mice used for analyzing the behavioral tests, mEPSCs, Golgi staining, immunostaining, and SUnSET tests.

(C) The detailed cohorts of *Ppp4r3a*-OE and control mice used for CUMS modeling and culturing of primary hippocampal neurons.

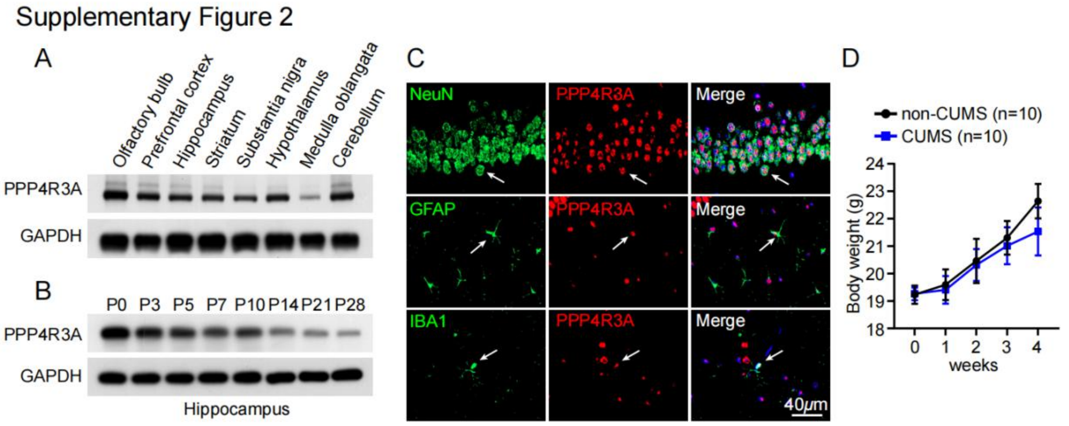

Fig. S2. The expression pattern of PPP4R3A in the central nervous system.

(A) PPP4R3A protein expression in different brain regions of adult WT mice (C57BL/6).

(B) PPP4R3A protein expression in the hippocampus during the early developmental period analyzed by Western blotting.

(C) Localization of PPP4R3A in neurons, astrocytes and microglia was verified by immunostaining of PPP4R3A with NeuN, GFAP and IBA1 using hippocampal slices, showing nuclear localization.

(D) The body weight of CUMS-exposed mice showed no difference with non-CUMS-exposed mice (two-way ANOVA, main effect of CUMS, $F_{1,90} = 0.7742$, $P = 0.3812$; interaction, $F_{1,16} = 0.2164$, $P = 0.6481$).

Disease Models & Mechanisms • Supplementary information

Supplementary Figure 3

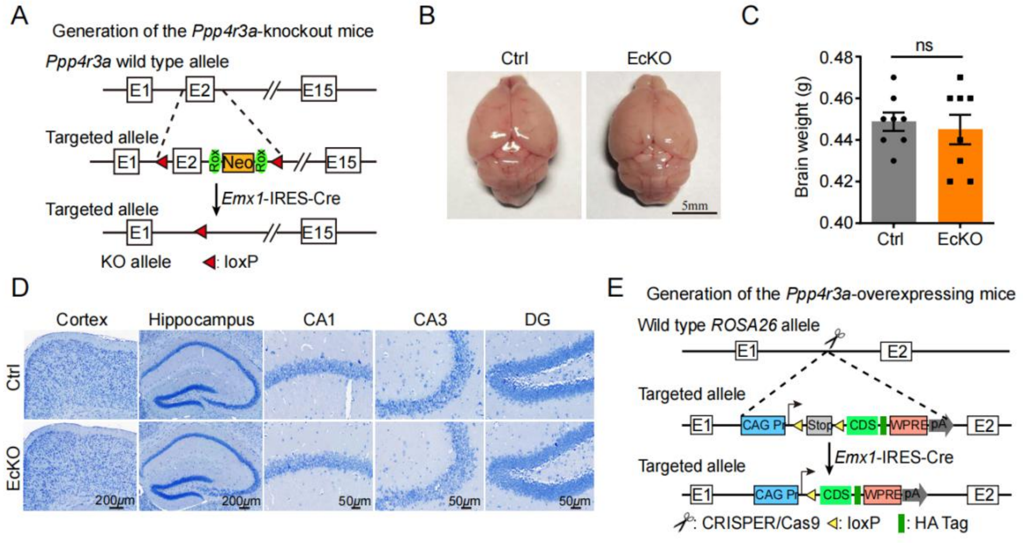

Fig. S3. Generation of *Ppp4r3a* knockout or overexpressing mice.

(A) The design of *Ppp4r3a*$^{flox/flox}$ mice and the strategy for generating EcKO mice.

(B) Representative brain images showing no apparent change in size.

(C) Quantification of brain weight showed no difference between genotypes.

(D) Representative images of Nissl staining showed no difference in cortical thickness or hippocampal size or number of neurons in EcKO mice.

(E) The design of the *ROSA26-Ppp4r3a*$^{flox/flox}$ mice and the generation strategy of conditional *Ppp4r3a*-OE mice.

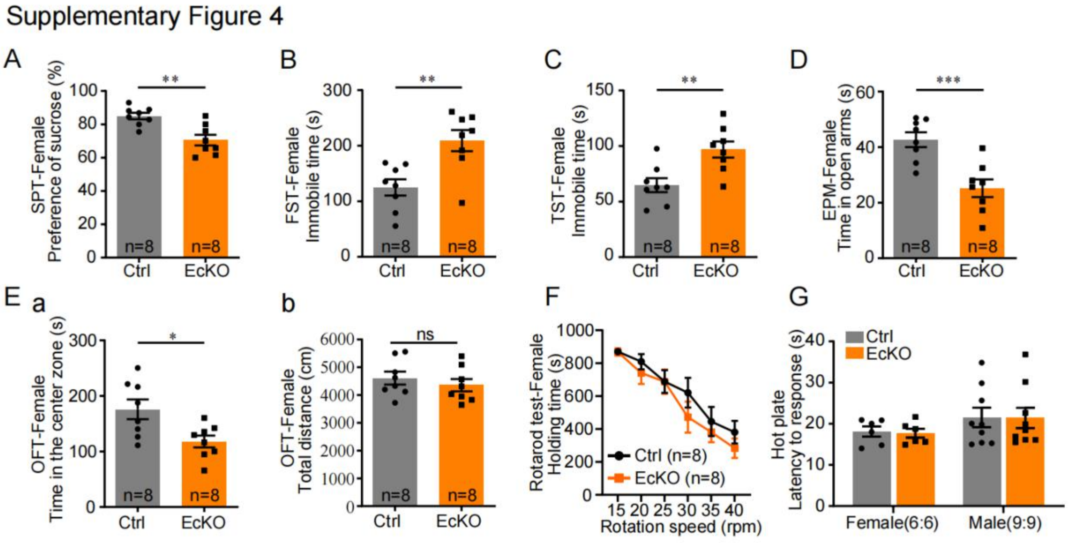

Fig. S4. The behavioral analysis of EcKO mice.

(A) Results of sucrose preference test (SPT). Female EcKO mice showed a decreased preference for sucrose compared with Ctrl mice (unpaired Student's *t*-test, t = 3.854, df = 14, P = 0.0018, Ctrl n = 8, EcKO n = 8).

(B) Results of forced swimming test (FST). Immobile time was increased in female EcKO mice compared with that in Ctrl mice (unpaired Student's *t*-test, df = 14, t = 3.513, P = 0.0034).

(C) Results of tail suspension test (TST). Immobile time was increased in female EcKO mice compared with that in Ctrl mice (unpaired Student's *t*-test, df = 14, t = 3.338, P = 0.0049).

(D) Results of EPM test. Time spent in the open arms was decreased in female EcKO mice (unpaired Student's *t*-test, df = 14, t = 4.221, P = 0.0009).

(E) Results of OFT. Decreased time spent in the center zone was showed in EcKO mice (a, unpaired Student's *t*-test, df = 14, t = 2.786, P = 0.0146); No difference in moving distance was showed in EcKO mice (b, unpaired Student's *t*-test, df = 14, t = 0.7984, P = 0.4379).

(F) Results of rotarod test. Normal motor endurance in holding the rotating rod in female EcKO mice compared with that in Ctrl mice (two-way ANOVA, main effect of genotype, $F_{1,90}$ = 2.692, P = 0.1050).

(G) Hot plate tests. No difference of withdrawal latency at temperature of 50 °C was showed in EcKO mice compared to control mice (two-way ANOVA, main effect of genotype, $F_{1,\ 26}$ = 0.01492, P = 0.9037).

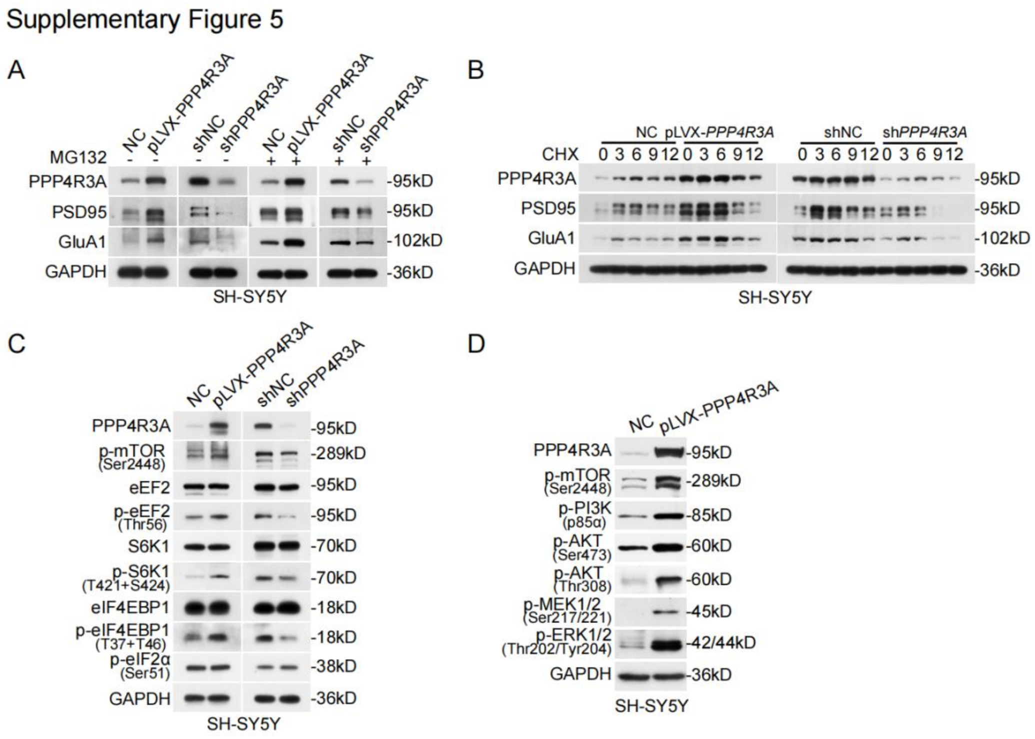

Fig. S5. *PPP4R3A* modulates expression of GluA1 and PSD95 in protein synthesis but not protein degradation way.

(A) Immunoblot analysis showed increased GluA1 and PSD95 in *PPP4R3A*-overexpressing cells and decreased GluA1 and PSD95 in *PPP4R3A*-knockdown cells with or without MG132 treatment. (a) Representative blotting bands; (b, c) Quantification of Western blot band intensity from 3 repeated tests (unpaired Student's *t*-test, ****P*<0.001, *****P*<0.0001).

(B) Immunoblot analysis showed normal degradation of GluA1 and PSD95 in *PPP4R3A*-overexpressing or *PPP4R3A*-knockdown SH-SY5Y cells with CHX administration.

(C) Immunoblot analysis showed positive regulation of PPP4R3A on mTORC1 signaling with *PPP4R3A*-overexpressing or *PPP4R3A*-knockdown SH-SY5Y cells.

(D) Immunoblot analysis showed positive regulation of *PPP4R3A* on ERK and PKB/ATK with *PPP4R3A*-overexpressing SH-SY5Y cells.

Supplementary Figure 6

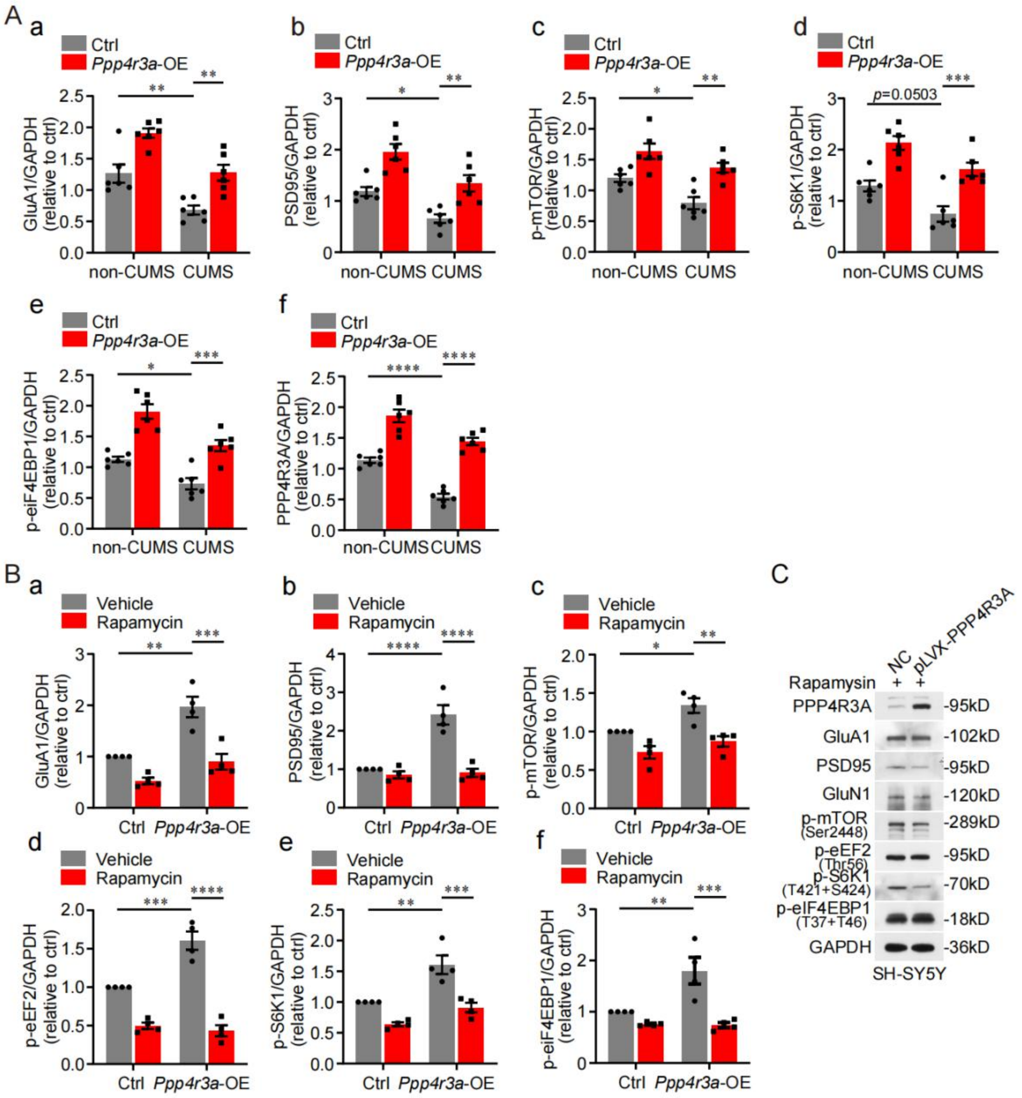

Disease Models & Mechanisms • Supplementary information

Fig. S6. *Ppp4r3a* overexpression in mice improves mTORC1 activity and protectsagainst chronic unpredictable mild stress (CUMS)-induced effects.

(A) Quantification analysis of western blot band intensity in Fig. 5G. (a) GluA1 (two-way ANOVA, main effect of genotype, $F_{1, 20} = 31.78$, $P < 0.0001$; main effect of CUMS, $F_{1,20} = 30.48$, $P < 0.0001$; interaction, $F_{1,20} = 0.05582$, $P = 0.8156$; Bonferroni *post hoc* test, *$P < 0.05$, **$P < 0.01$, ***$P < 0.001$, ****$P < 0.0001$); (b) PSD95 (two-way ANOVA, main effect of genotype, $F_{1, 20} = 33.6$, $P < 0.0001$; main effect of CUMS, $F_{1,20} = 20.54$, $P = 0.0002$; interaction, $F_{1,20} = 0.1149$, $P = 0.7382$); (c) p-mTOR (two-way ANOVA, main effect of genotype, $F_{1, 20} = 29.05$, $P < 0.0001$; main effect of CUMS, $F_{1,20} = 12.74$, $P = 0.0019$; interaction, $F_{1,20} = 0.5487$, $P = 0.4675$); (d) p-S6K1 (two-way ANOVA, main effect of genotype, $F_{1, 20} = 42.01$, $P < 0.0001$; main effect of CUMS, $F_{1,20} = 16.16$, $P = 0.0007$; interaction, $F_{1,20} = 0.01363$, $P = 0.9082$); (e) p-eiF4EBP1 (two-way ANOVA, main effect of genotype, $F_{1, 20} = 60.13$, $P < 0.0001$; main effect of CUMS, $F_{1,20} = 27.82$, $P < 0.0001$; interaction, $F_{1,20} = 0.7523$, $P = 0.3960$); (f) PPP4R3A (two-way ANOVA, main effect of genotype, $F_{1, 20} = 142.5$, $P < 0.0001$; main effect of CUMS, $F_{1,20} = 54.97$, $P < 0.0001$; interaction, $F_{1,20} = 1.682$, $P = 0.2094$).

(B) Quantification analysis of western blot band intensity in Fig. 5J. (a) GluA1 (two-way ANOVA, main effect of genotype, $F_{1, 12} = 35.20$, $P < 0.0001$; main effect of CUMS, $F_{1,12} = 22.61$, $P = 0.0002$; interaction, $F_{1,12} = 5.195$, $P = 0.0417$; Bonferroni *post hoc* test, *$P < 0.05$, **$P < 0.01$, ***$P < 0.001$, ****$P < 0.0001$); (b) PSD95 (two-way ANOVA, main effect of genotype, $F_{1, 12} = 32.85$, $P < 0.0001$; main effect of CUMS, $F_{1,12} = 25.67$, $P = 0.0003$; interaction, $F_{1,12} = 22.26$, $P = 0.0005$); (c) p-mTOR (two-way ANOVA, main effect of genotype, $F_{1, 12} = 26.86$, $P = 0.0002$; main effect of CUMS, $F_{1,12} = 11.35$, $P = 0.0056$; interaction, $F_{1,12} = 1.87$, $P = 0.1965$; (d) p-eEF2 (two-way ANOVA, main effect of genotype, $F_{1, 12} = 131.4$, $P < 0.0001$; main effect of CUMS, $F_{1,12} = 13.64$, $P = 0.0031$; interaction, $F_{1,12} = 21.01$, $P = 0.0006$); (e) p-S6K1 (two-way ANOVA, main effect of genotype, $F_{1, 12} = 36.94$, $P < 0.0001$; main effect of CUMS, $F_{1,12} = 25.48$, $P = 0.0003$; interaction, $F_{1,12} = 3.694$, $P = 0.0787$); (f) p-eiF4EBP1 (two-way ANOVA, main effect of genotype, $F_{1, 12} = 23.99$, $P < 0.0001$; main effect of CUMS, $F_{1,12} = 8.666$, $P = 0.0123$; interaction, $F_{1,12} = 9.479$, $P = 0.0096$).

(C) Immunoblot analysis showed that rapamycin treatment blocked the increased synaptic protein synthesis (GluA1, PSD95) in *PPP4R3A*-overexpressing SH-SY5Y cells by direct inhibiting mTORC1 signaling.

Disease Models & Mechanisms • Supplementary information

Table S1. CUMS procedure of mice.

Supplementary Table 1. CUMS procedure of mice.			
**Day of stress**	**Morning**	**Afternoon**	**Night**
1	9:00-11:00 physical restraint		19:00-7:00 cage tilt
2	9:00-12:00 overcrowding		19:00-7:00 water deprivation
3	7:00-9:00 empty bottle		19:00-7:00 wet cage
4	11:00-23:00 food deprivation		
5		14:00-14:45 cold exposure	19:00-22:00 cage exchange
6	7:00-19:00 dark		19:00-7:00 light
7		16:00-16:05 forced swimming	
8			19:00-7:00 food deprivation
9	9:00-12:00 overcrowding		19:00-7:00 wet cage
10	11:00-11:45 cold exposure		19:00-21:00 physical restraint
11			19:00-7:00 water deprivation
12	7:00-9:00 empty bottle		19:00-22:00 cage exchange
13	7:00-19:00 dark		19:00-7:00 light, cage tilt
14	11:00-23:00 food deprivation		
15		14:00-17:00 overcrowding	19:00-7:00 water deprivation
16	7:00-9:00 empty bottle		19:00-7:00 wet cage
17			19:00-19:45 cold exposure
18		14:00-16:00 physical restraint	
19	7:00-19:00 dark		19:00-7:00 light, cage tilt
20		14:00-14:05 forced swimming	
21	9:00-11:00 physical restraint		9:00-12:00 cage exchange
22			19:00-7:00 food deprivation
23	7:00-10:00 cage exchange		19:00-7:00 wet cage
24			20:00-22:00 physical restraint
25	7:00-19:00 dark		19:00-7:00 light, cage tilt
26	9:00-12:00 overcrowding		19:00-7:00 water deprivation
27	7:00-9:00 empty bottle	14:00-14:05 forced swimming	
28	11:00-11:45 cold exposure		19:00-21:00 physical restraint

Disease Models & Mechanisms • Supplementary information

Supplementary Materials and Methods

Antibodies and Reagents

Primary antibodies used in this study were as follows: PPP4R3A (abcam, ab70635, used in cellular experiments), PPP4R3A (Sigma, HPA002568, used in mice), FLAG (Sigma, F1804), GFAP (abcam, ab7260), NeuN (Millipore, MAB377), IBA1 (Proteintech, 10904-1-AP), GluA1 (Abways, CY5801, western blot), GluA1 (Affinity, AF6306, IF), PSD95 (Abways, CY5407, western bot), PSD95 (Affinity, AF5283), GluN1 (Abways, CY6798), GluN2A (Abways, CY6968), Synaptophysin (Abways, CY5273), vGlut1 (Abways, CY8055), vGAT1 (Abways, CY8146), Camk2a (Abways, CY6778), p-mTOR (Ser2448, CST, 5536S), p-S6K1 (T421/S424, Abways, CY5261), S6K1 (Abways, CY5324), p-eEF2 (Abclone, AP0832), eEF2 (Abways, CY7111), p-eIF2α (Abways, CY5036), p-eIF4EBP1 (Affinity, AF3830), eIF4EBP1 (Abways, CY6583), p-ERK1/2 (Thr202/Tyr203, CST, 4370T), p-MEK1/2 (Ser217/ Ser221, Affinity, AF8035), p-PI3K(p85α) (CST, 17366S), p-AKT (Ser473, CST, 4060T), p-AKT (Thr308, CST, 13038T), Puromycin (Millipore, MABE343), GAPDH (Proteintech, 60004-1-Ig). Secondary anti-rabbit or anti-mouse IgG Alexa Fluor® 488 or Alexa Fluor® 594 antibodies (1:500, Life Technologies). Other reagents include puromycin (Sangon Biotech, A610593), CHX (HY-12320, MCE), MG132 (A2585, ApexBio Technology).

Behavioral tests

Sucrose preference test (SPT)

Mice were housed individually and habituated to 1% sucrose solution for 2 days before the formal tests. For test, mice were given free access to two bottles containing 1% sucrose or water for 24h. Then, switching the positions of the two bottles to avoid bottle side preference. Sucrose preference (%) = sucrose consumption/ total fluid consumption × 100%. The average preference of the two days is the final number.

Forced swimming test (FST)

Each mouse was placed in a cylindrical glass breaker (25 cm in height, 16 cm in diameter) filled with 15 cm height of water (22 ± 0.5 °C). A video tracking system (Smart 3.0, Panlab, Spain) was used to record the movement parameters of the mice in water for 6 minutes. And the immobile time within 6 minutes was calculated to reflect depression degree of the mice. For formal testing, the

Disease Models & Mechanisms • Supplementary information

operator waited outside the behavioral testing room.

Tail suspension test (TST)

Clamping the tail of each mouse (1 cm from tip) and hanging it from the barb of a rectangular hanging box (15 cm in diameter, 30 cm in height) for 5 minutes. Using Smart 3.0 to record the immobile time within 5 minutes to reflect the depression degree.

Open field test (OFT)

Each mouse was placed in a lighting (100 lux) open field box made of light-grey polyvinyl chloridde (PVC, 40 × 40 ×40 cm), and was allowed to free explore for 10 minutes. Using Smart 3.0 to record the total time spent in the center zone, assessing for anxiety-like behavior.

Elevated plus maze (EPM)

The elevated plus maze consisted of four elevated arms (two 35 × 6 × 15 cm closed arms, and two 35 × 6 cm open arms), which radiated from a central platform (6 × 6 cm) to form a plus shape. The apparatus was arranged 60cm above the ground. Each mouse was placed into the center platform facing an open arm and was allowed to free explore for 5 minutes. Recording the time spent in open arms with Smart 3.0 to measure anxiety-like behaviors.

Barnes maze

The Barnes maze consisted of a circular platform (95 cm in diameter) with 20 holes (5 cm in diameter) equally spacing in the periphery, and was arranged 95 cm above the ground. One of the holes had a detachable acrylic black box under the hole entrance, enabling the mice to escape from lighting stimuli and return to their original cage (escape tunnel). To help the mice remember the position of the escape tunnel, setting reference subjects on the four walls outside the maze with different color and graphic (triangle, square, circle, pentacle). For the formal experiment, each mice was placed in the center of the maze covered by a non-transparent drum for 30 seconds for habituation. Then, the mouse was exposed to bright light and allowed free explore for 3 minutes to find the escape tunnel. From opening the drum, the Smart 3.0 software automatically recorded the moving path and time. The time that the mouse independently found the escape tunnel was recorded as its achievement. When the mouse got into the box, the operator immediately returned the mouse with the box to its original cage and release the mouse. If the mouse failed to find the box in 3 minutes, the operator gently guided it towards the box and returned it back to its original cage in the same way. And the escaping time was recorded as 180 seconds. The escape box maintained at same place during the test. Through successive

Disease Models & Mechanisms • Supplementary information

experiment, the mice would realize that finding the escape box meant a way to return the original cage so that they searched the box independently. A course of four days was used to assess the spatial learning memory of mice.

Fear conditioning test

Fear conditioning system is constructed based on Pavlovian conditioned reflex, using to measure contextual or cued fear memories and reflect anxiety-like degree of mice through their emotional reactivity in response to aversive stimuli (foot shock). The apparatus (FreezeScan, Cleversys Inc.) used in this experiment include two test chambers (32 × 26 × 21 cm), the ceiling, front and back of which were transparent and the floor of which was made of a removable grid of stainless steel rods. The first day was conditioning training, allowing the mice to obtain fear memories. First, each mice was placed in the chamber for 3 minutes for habituation. Then, a vocal cue (3kHz, 80db) was administered for 30 seconds, and a 2-second electric shock (0.6mA) was administered at the last 2 seconds of the sound stimuli. A total of 3 shocks were given with each interval of 1 minutes. The freezing time in response to startle stimuli was recorded reflecting baseline emotional reactivity of mice. The second day was a two-session test for short-term fear memories. The first stage was contextual-induced memory. Each mouse was placed in the chamber for 5 minutes without stimulation, and the freezing time was recorded. The second stage (sound-induced memory) was conducted after 1h in a new compartment. Each mice was placed in a bright condition to free explore for 3 minutes. Then, a vocal cue (3kHz, 80db) maintained for 180 seconds was administered, and the freezing time was recorded.

Hot plate test

Using the Ugo Basile hot plate model 35100 (Columbus Instruments) to assess the withdrawal latency of mice, commonly used for measuring sensory difficulties. Before the test, mice were habituated to the testing room for 30 minutes. For testing, each mice was placed in a Plexiglas cylinder on the surface of a hot plate at a temperature of 50°C. The latency of foot withdrawal was recorded, including jumping, lifting or licking hind paws. Three repeated trials on each mice were performed at least half an hour apart.

Miniature excitatory postsynaptic currents (mEPSCs)

Postnatal 8-week-old EcKO mouse and their Ctrl littermates were prepared for mEPSCs tests. After the mouse was deeply anesthetic with urethane (25%, 1ml/100g), the brain was isolated rapidly

Disease Models & Mechanisms • Supplementary information

and transferred to 0-4°C artificial cerebrospinal fluid (ACSF, consisted of 234 mM Sucrose, 2.5 mM KCl, 1.25 mM $NaH_2PO_4 \cdot 2H_2O$, 25 mM $NaHCO_3$, 25 mM D-Glucose, 0.5 mM $CaCl_2 \cdot 2H2O$, 10 mM $MgSO_4$) bubbled with 95% O_2 and 5% CO_2. In the culture dish filled with cold ACSF, the cerebellum, forebrain and the brainstem of mouse were removed. The remaining tissue containing hippocampus was prepared for coronal slicing (300 μm thick) using a vibrating slicer (Leica, VT 1000 S, Germany). The prepared slices were immediately transferred to ACSF incubation buffer (125 mM NaCl, 2.5 mM KC, 1.25 mM $NaH_2PO_4 \cdot 2H_2O$, 25 mM $NaHCO_3$, 10 mM D-Glucose, 2mM $CaCl_2 \cdot 2H_2O$, 1.5 mM $MgSO_4$) at 32°C and incubated for 30 minutes.

For recording, the brain slices were placed in a perfusion slot filled with incubation buffer, and 1μM TTX and 10 μM Bicuculline were added to the buffer which was continually bubbled with 95% O_2 and 5% CO_2. The temperature was maintained at 31±1ºC. The voltage stimulus protocol for recording mEPSCs is as follows: the clamping voltage is -70 mV. Form a high resistance sealing pre-compensation offset pre-offsetting, record the electrode close to the cell and give the cell negative pressure to form a high resistance seal (>1 GΩ), seal after rapid capacitive compensation. Record a 5mV voltage stimulation (test series resistance) after the negative pressure breaks the membrane and waits for the cell to stabilize 3-5 min at the -70mV interface. Switch to gap-free mode and record 10 min as a blank group after stabilization. The model group recording is 10 min stable for the dosing group. Record changes in the series resistance before and after the test. The experimental datas were offline analyzed using Clampfit 10.6 and GraphPad 8.0.2 to calculate the current amplitude and discharge frequency, reflecting the presynaptic glutamate release and expression of postsynaptic receptors.

Golgi staining

Golgi staining was performed using the TM FD Rapid GolgiStain Kit (FD NeuroTechnologies, MD, USA). Each mouse was deeply anesthetized with 60mg/kg sodium pentobarbital. The fresh brain was quickly isolated and immersed in the impregnation solution for 2 weeks in the dark at room temperature. Then, the mouse brain was transferred to Solution C and stored for at least 48 hours in the dark at 4°C. After, brain slices were cut at room temperature (100μm thick) using a vibratome (VT1200S, Leica, Germany). Slices were stained with Solution D and Solution E for 5-10 minutes at 4°C followed by ddH_2O rinse for 3 times. Then, the slices were dehydrated in successive baths of ethanol, cleared with Histoclear, and cover-slipped with DPX mounting medium. The images were

captured using the 3DHISTECH microscope with z stack at 20 (Budapest, Hungary) and were analyzed with CaseViewer 2.0 Software (3DHISTECH, Budapest, Hungary).

SUnSET

Primary hippocampal neurons were obtained from the embryos at embryonic day 18.5 (E18.5) mated by *Ppp4r3a*-OE mice with ROSA26-*Ppp4r3a*$^{flox/flox}$ mice. In pre-cooled HBSS, the hippocampus of embryos was isolated and mechanically shredded to about 1mm^3 size, digested with 0.05% trypsin-EDTA and terminated digestion with DMEM/F12 containing 10%FBS. The cells were gently swirled with neurobasal medium supplemented with 2% B27 and 0.1mg/ml L-glutamine, and seeded on coverslips or six-well plates precoated with 0.1mg/ml poly-L-Lysine after filtration with a 0.22um filter. Neurons were cultured at 37°C in an incubator with 5% CO_2. For SUnSET tests, cultured primary hippocampal neurons were administered with 10ug/ml puromycin for 30 minutes at 37°C and then incubated in fresh medium for 3h. Then, the cells were collected with RIPA lysates to extract the proteins for western blotting analysis with Puromycin antibody.

EcKO and littermate WT mice were used for *in vivo* analysis of protein synthesis. Each mouse was anesthetized with an isoflurane vaporizer (1.5%) and mounted in a standard stetrotaxic instrument (No: 68043, RWD Life Science Inc., Shen Zhen, China). After disinfecting with iodophor, an incision was made in the scalp to exposure the brain, which was leveled based on the horizontal positions of the bregma and lembda. Then, the punch position of the lateral ventricle (AP: -0.2; ML: -1.0; DV: -2.4) was determined for puromycin microinjection. The puromycin (10ug/ml) was injected at a rate of 0.4ul/min for a total volume of 2.5ul. After 1h of injection, the mouse was dissected to isolate PFC and hippocampus tissues respectively. Then, the total protein synthesis in PFC or hippocampus was measured with western blotting analysis.

Plasmid Construction

The wildtype *PPP4R3A* expression vector was constructed using the full length of human *PPP4R3A* coding sequence (ENSG00000100796.17), which was cloned into pLVX-IRES-puro vector. And the vector of pLVX-IRES-puro or pLVX-*PPP4R3A* was used to infect SH-SY5Y cells, leading to generation of *PPP4R3A* overexpressing and control cell lines. In addition, the GV248 lentiviral vector containing sh*PPP4R3A* (ACAACAGAATGATGATGAT) or scramble (TTCTCCGAACGTGTCACGT) sequence was used for generation of *PPP4R3A*-deficient and control cells by transfection to SH-SY5Y cells. To figure out the positive regulation of *PPP4R3A* on synaptic proteins were through increased

Disease Models & Mechanisms • Supplementary information

protein synthesis or decreased degradation, the cells were used for analyzing the expression of GluA1 and PSD95 with treatment of CHX or MG132.

Western blot analysis

The tissues or collected cells were extracted using strong RIPA lysis buffer (P0013C, Beyotime Biotechnology) with two kinds of 1% protease inhibitor cocktail (K1007 and K1015, ApexBio Technology). The lysates were denatured with SDS loading buffer to run 10% SDS-PAGE. After, transferring the proteins onto PVDF membranes (Merck Millipore, Germany), incubating the membrane with 5% nonfat milk, and analyzing the protein expression with specific antibodies.

Primers

Genotyping primers for *Ppp4r3a* overexpressing mice

Forward primer 1: 5'-AGTCGCTCTGAGTTGTTATCAG-3'

Reverse primer 1: 5'-TGAGCATGTCTTTAATCTACCTCGATG-3'

Reverse primer 2: 5'-GTCAATGGAAAGTCCCTATTGGCGT-3'

The 469bp alone product was from WT mice, the 278bp alone product was from homozygous *Ppp4r3a*-floxed mice, and the product with two bands was from heterozygous mice.

Genotyping primers for conditionally *Ppp4r3a* knockout mice

Forward primer 1: 5'-ATTGCTTCCCAGTCTTCACTCTTC-3'

Reverse primer 1: 5'-TCCCAAAACCACCACAGAACTAAC-3'

The 296bp alone product was from WT mice, the 352bp alone product was from homozygous KO mice, and the product with two bands was from heterozygous mice.

Genotyping primers for *Cre* mice

Cre forward Primer: 5'-CATATTGGCAGAACGAAAACGC-3'

Cre reverse Primer: 5'-CCTGTTTCACTATCCAGGTTACGG-3'

The positive product size was 413bp.

Disease Models & Mechanisms • Supplementary information
